# Glucocorticoid ultradian rhythmicity differentially regulates mood and resting state networks in the human brain: A randomised controlled clinical trial

**DOI:** 10.1016/j.psyneuen.2020.105096

**Published:** 2021-02

**Authors:** Konstantinos Kalafatakis, Georgina M. Russell, Stuart G. Ferguson, Meryem Grabski, Catherine J. Harmer, Marcus R. Munafò, Nicola Marchant, Aileen Wilson, Jonathan C. Brooks, Jamini Thakrar, Patrick Murphy, Ngoc J. Thai, Stafford L. Lightman

**Affiliations:** a Laboratories of Integrative Neuroscience and Endocrinology, School of Clinical Sciences, University of Bristol, BS1 3NY Bristol, United Kingdom; b Clinical Research and Imaging Centre, University of Bristol and University Hospitals Bristol NHS Foundation Trust, BS2 8DX Bristol, United Kingdom; c School of Medicine, University of Tasmania, Hobart, TAS 7000, Australia; d Clinical Psychopharmacology Unit, Division of Psychology and Language Sciences, University College London, WC1E 6BT London, United Kingdom; e MRC Integrative Epidemiology Unit, School of Psychological Science, University of Bristol, BS8 1TU Bristol, United Kingdom; f Department of Psychiatry, Oxford University and Oxford Health NHS Foundation Trust, OX3 7JX Oxford, United Kingdom; g Royal Bristol Infirmary, University Hospitals Bristol NHS Foundation Trust, BS2 8HW Bristol, United Kingdom

**Keywords:** Glucocorticoids, Ultradian rhythm, FMRI, Mood, Psychophysiology

## Abstract

Adrenal glucocorticoid secretion into the systematic circulation is characterised by a complex rhythm, composed of the diurnal variation, formed by changes in pulse amplitude of an underlying ultradian rhythm of short duration hormonal pulses. To elucidate the potential neurobiological significance of glucocorticoid pulsatility in man, we have conducted a randomised, double-blind, placebo-controlled, three-way crossover clinical trial on 15 healthy volunteers, investigating the impact of different glucocorticoid rhythms on measures of mood and neural activity under resting conditions by recruiting functional neuroimaging, computerised behavioural tests and ecological momentary assessments. Endogenous glucocorticoid biosynthesis was pharmacologically suppressed, and plasma levels of corticosteroid restored by hydrocortisone replacement in three different regimes, either mimicking the normal ultradian and circadian profile of the hormone, or retaining the normal circadian but abolishing the ultradian rhythm of the hormone, or by our current best oral replacement regime which results in a suboptimal circadian and ultradian rhythm. Our results indicate that changes in the temporal mode of glucocorticoid replacement impact (i) the morning levels of self-perceived vigour, fatigue and concentration, (ii) the diurnal pattern of mood variation, (iii) the within-network functional connectivity of various large-scale resting state networks of the human brain, (iv) the functional connectivity of the default-mode, salience and executive control networks with glucocorticoid-sensitive nodes of the corticolimbic system, and (v) the functional relationship between mood variation and underlying neural networks. The findings indicate that the pattern of the ultradian glucocorticoid rhythm could affect cognitive psychophysiology under non-stressful conditions and opens new pathways for our understanding on the neuropsychological effects of cortisol pulsatility with relevance to the goal of optimising glucocorticoid replacement strategies.

## Highlights

• Changes in the glucocorticoid rhythm impact morning levels of vigour, fatigue and concentration.
• They affect the diurnal variation of mood.
• They modulate the neural dynamics of large-scale resting state networks of the human brain.
• They could alter the functional relationship between mood variation and underlying neural networks.

## 1 Introduction

Adrenal glucocorticoid secretion into the systematic circulation is characterised by a complex rhythm, composed of a circadian variation, with hormonal levels rising to a diurnal peak during the active part of the day followed by a gradual decrease down to the diurnal trough. Underlying is an ultradian rhythm formed by short-lasting pulses of adrenal glucocorticoid secretion whose amplitude varies in a time-of-day-dependent manner (Russell et al., 2015). The characteristics of these pulses (duration, amplitude, and frequency) depend on many variables including genes, early life experience, age, gender and both internal and external stressors (Kalafatakis et al., 2016a).

In rodents, the diurnal glucocorticoid variation affects the expression of many genes (McEwen et al., 2015), regulates dendritic spine formation (Liston et al., 2013) and impacts emotional behaviour (Ikeda et al., 2013). Moreover, ultradian rhythmicity differentially regulates steroid-sensitive gene expression patterns in the hippocampus (Conway-Campbell et al., 2010), prefrontal cortex and pituitary (George et al., 2017). It also modulates synaptic plasticity (Sarabdjitsingh et al., 2014), dynamically defines periods of high and low neuroendocrine responsivity to stress (Sarabdjitsingh et al., 2010), influences the electrical activity patterns in the amygdala, modifying the efficacy of cognitive processes like fear learning (den Boon et al., 2019), and defines the context in which other neural stimuli with shared molecular downstream pathways (like the brain-derived neurotrophic factor) exert their effect upon brain cells (Huzard et al., 2020). The molecular basis for these rhythm-related biological effects lies on the different binding affinities of the two types of corticosteroid receptors with glucocorticoids, resulting in a differential activation during the oscillatory activity of the hormone, leading consequently to changes of genomic, epigenomic and postgenomic cellular events (de Kloet et al., 2018, Kalafatakis et al., 2019).

Does glucocorticoid ultradian rhythmicity affect the human brain function as well? The answer to this question is unclear. Until very recently, no direct data had been presented on the potential neurobiological significance of glucocorticoid pulsatility in human, as well as its clinical implications. Functional neuroimaging studies have observed either acute of delayed effects after hydrocortisone administration on the neural activation patterns of various corticolimbic regions (such as the prefrontal cortex, insula, amygdala or hippocampus) in the context of mobilising different cognitive mechanisms, like working memory, emotional processing, attentive processing, learning, or even resting state conditions (Henckens et al., 2010, Henckens et al., 2011, Henckens et al., 2012a, Henckens et al., 2012b, Henckens et al., 2012c, Symonds et al., 2012, Vaisvaser et al., 2013, Veer et al., 2012, Vogel et al., 2015a, Vogel et al., 2015b). Psychological studies have also demonstrated temporal differences in subjects’ reward-related decision making behaviour following hydrocortisone administration (Riis-Vestergaard et al., 2018). Moreover, stress induction studies, modifying endogenous systemic cortisol levels, also provide evidence for glucocorticoid time-dependent effects on neural activation patterns related to memory processing and learning (Vogel et al., 2017, Sep et al., 2019). Finally, observational studies suggest that cortisol ultradian dysrhythmicity may be causally involved in disorders like chronic insomnia (Vargas et al., 2018).

Following the hypothesis that glucocorticoid ultradian rhythmicity affects the human brain function, we have conducted a clinical trial on healthy volunteers, investigating the impact of different glucocorticoid rhythms on neural activity, measured by functional neuroimaging, and key markers of mood, cognition and behaviour. Pharmacological manipulation was used to suppress subjects’ endogenous glucocorticoid rhythm and we infused exogenous cortisol with predetermined rhythms. The outcome measures in the clinical trial were classified into two main categories: (i) measures of emotional processing, that we presented earlier (Kalafatakis et al., 2018), and (ii) measures of mood and neural activity under resting conditions, which we are presenting in this paper.

The outcome measures used in this part of the clinical study (resting state functional neuroimaging, ecological momentary assessment) represent exploratory analyses which provide clinical hypotheses for the future design of mechanistically oriented clinical trials to detail the role of glucocorticoid rhythmicity in human brain function.

## 2 Materials and methods

### 2.1 Introductory remarks

This was a randomised, double-blind, placebo-controlled, three-way crossover study registered with the United Kingdom Clinical Research Network (IRAS reference 106181, UKCRN-ID-15236; October 23, 2013). Each subject participated into three 5-day long study arms. Block randomisation schedules were generated by staff members not involved in data collection; each subject was randomly assigned to one of the six possible orders of treatments (Fig. S1). Dispensing and processing of all related products was managed by Bristol Royal Infirmary University Hospital Pharmacy. In each study arms, subjects were required to take the same daily regimen of tablets and remain connected to a subcutaneous infusion pump. Anonymized data from all outcome measures were stored in the University of Bristol central servers, were cleaned at an individual level without knowledge of which session corresponds to which subject, and, consequently, further postprocessed and compared statistically at a group level, without knowledge of which group corresponds to which mode of hydrocortisone substitution. Sample size was estimated based on the results of previous trials using similar neurobehavioural measures.

### 2.2 Study design

We recruited right-handed, English-speaking, healthy male individuals, 18–60 years of age, who did not smoke or smoked less than 6 cigarettes per day, drank less than 21 units of alcohol per week, and less than 4 caffeinated drinks per day. They had not participated in any other investigational trial in the last 2 months or were taking any kind of medication (prescribed, over the counter, recreational, including topical steroids and inhalers). Additionally, we excluded shift workers, or subjects who were dyslectic, claustrophobic or had any other contraindications for entering the scanner bore. Participants were kept extensively informed on the study goals, methods, safety issues and procedures. All appropriate measures, to ensure participants’ safety, were planned in advance (no such incidences occurred) and any side effects related to the treatment schedules recorded. The study also implemented a protocol for dealing with incidental findings in brain imaging research (no such incidental findings occurred). Finally, based on earlier clinical work in similar experimental settings, using neuropharmacological challenge to probe psychological mechanisms of mood regulation and emotional processing, and functional neuroimaging to measure related neural activity (Harmer et al., 2004, Browning et al., 2007, Murphy et al., 2008), we performed power calculations that confirmed a sample size of 15 participants per treatment arm, on whom complete data were available, would be required to give our study a 80% power at an alpha level of 5%. Further details on the study design and its rationale and limitations can be found in the study protocol, published elsewhere (Kalafatakis et al., 2016b).

The study was discussed with the Medicines and Healthcare products Regulatory Authority (MHRA) who agreed no formal application was necessary. The study was conducted according to the principles of the Research Governance Framework for Health and Social Care and the international conference for harmonisation of good clinical practice (ICH GCP, declaration of Helsinki). The Ethics Committee of the University of Bristol approved the study (Ethics approval code: 2706132525), additionally to the local Research and Development (R&D) approval of the University Hospitals Bristol (UHB, National Health System Foundation Trust) (reference code: ME/2013/4325), and all participants provided written informed consent.

### 2.3 Participants and interventions

Fifteen volunteers between 20 and 33 years of age were included in the study (Table S1) (Kalafatakis et al., 2018). The subjects had no history of neuropsychiatric disease as confirmed by clinical assessment and were excluded if they had received a diagnosis or had a first-degree family history of a psychiatric disorder. Each volunteer passed a detailed screening session, part of which was the acquisition of a high-resolution anatomic MRI scan (Fig. S1). The cortisol biosynthesis blocking agent metyrapone was taken orally in all three arms of the study to render them hypoadrenal (per 5-day study arm: 250 mg at lunch and 250 mg before going to sleep on the first day, 250 mg at breakfast, 500 mg at lunch and 500 mg before going to sleep on the second day, 500 mg at breakfast and lunch and 750 mg before going to sleep on the third day, 750 mg at breakfast and lunch and 1000 mg before going to sleep on the fourth day, and finally 750 mg at breakfast on the last day). Glucocorticoid replacement of the same total daily dose (20 mg) was administered via three different methods: (i) subcutaneous continuous hydrocortisone delivery via a pump (Animas® Vibe™ Insulin Pump), with a time-of-day varying rate (2 mg/h between 02:00–08:00, 1 mg/h between 08:00–12:00, 0.4 mg/h between 12:00–20:00 and 0.1 mg/h between 20:00–02:00), creating a normal circadian but no ultradian rhythmicity (SCC group), (ii) subcutaneous hydrocortisone delivery via a pump (Crono P®, CANE Applied Medical Technology Ltd, Cambridge, UK), delivering pulses of hydrocortisone every 3 h with amplitude varying in a time-of-day-dependent manner (4 mg at 03:00, 06:00 and 09:00, 2.3 mg at 12:00, 15:00, 18:00, and 0.5 mg at 21:00 and 00:00, with a rate of 0.1 mg/sec), creating the normal circadian and ultradian rhythm (SCP group), and (iii) *per os* intake of hydrocortisone pills, three times daily (10 mg after waking up, and 5 mg at lunch and dinner), creating a suboptimal circadian and ultradian cortisol rhythm, formed by long-lasting pulses with inconsistent interpulse intervals (PO group).

### 2.4 Ecological momentary assessment (EMA) on mood and fatigue

Throughout each 5-day treatment period (Fig. S2), subjects had to carry an android mobile phone running custom EMA software. The device was programmed to prompt participants to complete three different types of surveys: a daily morning report (to be completed by wake up until 10 am), a daily evening report (to be completed between 7 pm and midnight) and 5–6 surveys randomly spaced over the waking day (random prompts). The morning report included 29 statements contained in the Identity Consequences Fatigue Scale (ICFS). The morning report also included 9 items about self-perceived reactivity and feeling of wellbeing in the form of a visual analogue scale (VASQ) (Fig. S3). These 9 items were also answered during the evening report, and during the 5–6 random prompt surveys administered each day. Mood was assessed at random times, because it varies continuously over the course of the day and is not easily conceptualised in an episodic framework.

### 2.5 Emotionally-valenced, self-referral word categorisation task (ECAT)

This task was administered on the last day of each treatment arm (Fig. S2). It assesses the speed of responding to twenty positive and twenty negative self-referent personality descriptors, which were selected to be extremely disagreeable (e.g., boring, untidy, hostile) or agreeable (helpful, honest, optimistic). The descriptors were presented on the computer screen for 1000 ms, with the inter-stimulus interval (time between the disappearance of a previous word and the appearance of the next one) being 5000 ms. These words were matched in terms of word length and ratings of frequency and meaningfulness. Participants were asked to categorise these personality traits as likable or dislikable as quickly as possible. Specifically, they were asked to imagine whether they would be pleased or upset if they overheard someone else referring to them as each descriptor displayed. Participants were encouraged to make no response if they didn’t understand the meaning of a particular word, and just wait for the next one. Correct classifications in the form of %accuracy ([1 – [(N of wrong + N of non-responses)/Total N of trials]] * 100) and reaction times for correct identifications were computed for this task.

### 2.6 ECAT-related free recall task (EREC)

This task was administered on the last day of each treatment arm (Fig. S2). It is a surprise free recall task to assess the incidental encoding of the emotionally-valenced personality descriptors presented during the ECAT. About 10 min after the completion of ECAT, volunteers were given a piece of paper and a pen and asked to recall and write down as many of the personality descriptors from the ECAT (see above) as possible within a 2-min time window. The relative recall of positive versus negative words gives a measure of emotional biases in memory.

### 2.7 Statistical analyses of the behavioural data

Statistical analysis of the EMA data was performed by STATA® release 14. Over the course of the study, 162 ICFS and 1018 VARQ assessments were completed. Their number did not differ between conditions or across study days. As the strength of metyrapone-induced adrenal suppression reached its maximum level after the second day per study treatment [i.e. the glucocorticoid rhythm was minimally affected by any endogenous adrenal activity and based almost solely on the distinctively different patterns of exogenous administration, see Kalafatakis et al. (2016b) we only retained data from days 3–5 for further analysis. Moreover, in relation to the VASQ assessments, it was imperative to have complete datasets from different timepoints across each day to be able to model any mood oscillations throughout the day or across days. Therefore, we also excluded the few VASQ assessments issued in the morning of day 5, just before the end of the EMA study. Eventually, 118 ICFS and 503 VASQ assessments were used for the statistical analysis.

In order to explore the effect of different glucocorticoid rhythms on the self-perceived feeling of liveliness after waking up in the morning, five independent variables have been created from the ICFS data, according to the validated subscales used to score the ICFS (Paddison et al., 2006); VIGOUR, FATIGUE, DISTRACTION, MOTIVATION and ACTIVITY (Fig. S3). The effect of treatment condition on these five variables was estimated using an individual fixed effects model.

In order to explore the effect of different glucocorticoid rhythms on mood variation, principal component analysis was used to reduce the nine VASQ items and identify two factors: positive and negative affect (see Table S2). The effect of treatment condition on these two factors was also evaluated using an individual fixed effects model. Two approaches were used: (i) the effect of time over the 24-h period on the VASQ ratings was assessed for each treatment condition individually (circadian variation of mood) (Basabe et al., 2002) and (ii) the effect of day on the VASQ ratings was assessed for each treatment condition on each day individually for study days 3–4 (across-day evolution of mood).

Statistical analysis of ECAT and EREC data was performed using SPSS® version 23. The influence of the different cortisol rhythms on subjects’ response accuracy and reaction time was evaluated with a two-way, repeated-measures, mixed-model analysis of variance (ANOVA), with the one within-subject factor being treatment group (three levels: SCC, SCP, and PO) and the second being emotional valence (two levels: positive, negative). Tests for detecting outliers, normality in the distribution of data (Shapiro–Wilk test), and sphericity (Mauchly’s test) have been used and taken into consideration for the data analysis. Although the assumption of sphericity was not violated in any case, we used Greenhouse–Geisser correction regardless. Two-tailed tests were performed for all analyses, and P was set to 0.05. All results shown in the corresponding table are mean ± SD. Confidence intervals (CIs) refer to ± 2 SD. As a measure of the effect size, ω^2^ was used for the ANOVA. Pairwise comparisons with Bonferroni adjustment were performed to investigate any (simple) main effects of treatment across the three study groups.

Two subjects were systematically performing poorly in the verbal-dependent behavioural tests (the studentized residuals of the %accuracy scores had values lower than − 3). Both participants were non-native English speakers (coming from Latin America and Africa respectively); the low scores reflect their occasional limited understanding of the meaning of the personality descriptors, for most of which they’ve chosen to respond (although instructed otherwise) randomly. This is a systematic bias which needs to be removed; both subjects were therefore excluded from data analysis.

### 2.8 Resting state functional neuroimaging study

The functional neuroimaging study was performed on the last day of each treatment arm (Fig. S2). Its technical aspects are described in Kalafatakis et al. (2016b). Functional neuroimaging data analyses was carried out using FMRIB software library (FSL) 5.0 (Jenkinson et al., 2012), and involved various preprocessing, postprocessing and validation steps (Fig. S4). Briefly, these steps consisted of (i) brain intensity normalisation, (ii) 3D motion correction (by using FSL linear registration tool, MCFLIRT), (iii) B_0_ unwarping, (iv) brain extraction, (v) spatial smoothing, (vi) temporal high pass filtering and (vii) co-registration of functional images with corresponding high-resolution anatomical images (by using field maps acquired prior the fMRI experiment) and the latter with MNI152 standard space.

### 2.9 Strategy for analysing the resting state neuroimaging data

We used three independent approaches to utilise the resting state functional neuroimaging data (Fig. S4). The first of them is totally exploratory, implementing independent component analysis (ICA) at individual and group-level, data denoising and isolation of group-level resting state networks (RSNs) of the human brain, then, for each subject, estimation of a "version" of each of those group-level RSNs by regressing the group-RSNs into each subject's 4D dataset to give a set of time courses and subsequently regressing those time courses into the same 4D dataset to get a subject-specific set of RSNs, and finally comparing these RSNs across groups of subjects to look for group differences. This process is commonly used in the analysis of resting state functional neuroimaging data (Zuo et al., 2010, Li et al., 2018).

For the other two approaches we performed a hypothesis-driven, seed-based analysis (rather than an exploratory one across the whole brain). We based our hypotheses on three main concepts: (i) brain regions mainly susceptible to glucocorticoid effects (due to the presence of glucocorticoid and mineralocorticoid receptors) belong to the corticolimbic system of the brain (amygdala, striatum, hippocampus, insula, cingulate, orbitofrontal cortex) (Kalafatakis et al., 2016a), which is crucial for regulating emotional processing and mood, (ii) particularly in the context of positive mood regulation and brain reward circuitry dysregulation in mood disorders (Russo and Nestler, 2013), amygdala, orbitofrontal cortex and ventral striatum (nucleus accumbens) play an important role (Namburi et al., 2016), as clinical (Nitschke et al., 2004) and preclinical (Burgdorf and Panksepp, 2006) sources of evidence suggest, (iii) there are three widely recognised large-scale RSNs of the human brain (default-mode network/ DMN, salience network/ SN, executive control network/ ECN), which either contain or interact with parts of the corticolimbic system, and whose neural dynamics are affected by glucocorticoid- or stress-related mechanisms (Soares et al., 2017, Taren et al., 2017, Peters et al., 2016, Thomason et al., 2011), and (iv) in our previous report (Kalafatakis et al., 2018), we were able to show that various parts of the right-sided corticolimbic system show differential neural responses in a glucocorticoid rhythm-dependent manner.

Taking the previously mentioned concepts into consideration, we hypothesised (i) that regions of the right-sided corticolimbic system might change their functional connectivity with the default-mode network, salience network and executive control network depending on the underlying glucocorticoid rhythm, and (ii) that positive mood variation might relate to different neural network dynamics of the right amygdala, right orbitofrontal cortex and right nucleus accumbens in the different treatment groups (Fig. S5).

### 2.10 Statistical analyses of the neuroimaging data

Probabilistic ICA was carried out using a subcomponent of FSL (MELODIC) (Beckmann and Smith, 2004), in two stages; at individual level, independent component maps (IC maps) have been created per subject and resting state fMRI session with spatial ICA. The IC maps were thresholded using an alternative hypothesis test based on fitting a Gaussian/gamma mixture model to the distribution of voxel intensities within spatial maps and controlling the local false-discovery rate at p < 0.5. A data denoising system was used based on visual inspection of IC maps as described in Kelly et al. (2010). Intensity normalisation across volumes of fMRI data was performed, to filter out signals that affect the entire brain simultaneously. After removing the noisy components from the original 4D dataset, four group ICAs have been performed (one including all sessions from all treatment groups, and one per combination of two treatment groups), each followed by another denoising session.

The three main large-scale RSNs of the human brain (DMN, SN, ECN) (Menon, 2011) have been isolated in MNI152 standard space from the denoised output of the across-treatments group ICA, and the corresponding regions were subsequently estimated individually for each session and each participant in the resting state functional images by reversing the spatial transformations that have been applied by MELODIC during the corregistration of functional images to the corresponding high-resolution images to MNI152 standard space, and timeseries extraction has been applied. These timeseries have been used in the correlation analysis with regions of interest (ROIs, see below).

The analysis for the differences between each of the three combinations of treatment groups was carried out using the FSL dual regression technique that allows for voxel-wise comparisons of resting-state fMRI (Filippini et al., 2009, Veer et al., 2010). This involves using the group-ICA spatial maps in a linear model fit against the separate fMRI data sets, resulting in time-course matrices describing the temporal dynamics for each component and subject, and then using these time-course matrices to estimate subject-specific spatial maps. The dual regression analysis was performed with variance normalisation. In this context, the dual regression reflects differences in both activity and spatial spread of the RSNs. The different component maps have been tested voxel-wise (paired *t*-test model per combination of treatment groups) for statistically significant differences using FSL Randomize nonparametric permutation testing, with up to 10,000 permutations, correcting for multiple comparisons voxel-wise.

Aside the probabilistic approach, we intended to compare the strength of correlation between predefined ROIs with DMN, SN and ECN, between the three treatment groups. We’ve used the Harvard-Oxford subcortical structural atlas in MNI152 standard space to estimate our seeds (see Table S3) (Yan et al., 2009). After seed generation in MNI152 standard space, the corresponding seed region was estimated individually for each session and each participant in the resting state functional images, using the original seed created and reversing the spatial transformations that have been applied by MELODIC during the corregistration of functional images to the corresponding high-resolution images to MNI152 standard space, and timeseries extraction has been applied per ROI, subject and treatment arm. Each of those timeseries has been tested against the corresponding timeseries of the three large-scale RSNs by Spearman’s rank-order correlation analysis at individual level, and with Fisher z-transformation for group-level pairwise comparisons. Level of significance has been set to 0.05. SPSS® version 23 and MedCalc® have been used for this purpose.

Finally, we wanted to identify certain ROI-dependent, resting state networks, whose within-treatment group, between-subject variance in the strength of their functional connectivity/ dysconnectivity correlates significantly with the corresponding variance in the EMA data referring to factor positive affect, acquired a few minutes prior to the MRI scan. The ROI-based functional connectivity analysis was performed following the methodology proposed by (Di Martino et al., 2008), after being validated (Fig. S5). For the correlation analysis, a whole-brain, within-group mixed effects model, implemented in FSL (FLAME), was used. Nine separate correlation analyses were performed (3 treatment groups × 3 seeds). Corrections for multiple comparisons were performed at the cluster level using Gaussian random field theory (minimum z > 2.3, cluster p threshold < 0.05). These group-level analyses produced thresholded z score maps highlighting resting state networks, functionally associated/ dissociated from each ROI, that have a linear positive relationship with the EMA-derived scores reflecting positive affect per treatment group. Inherent to the process, correction for multiple comparisons has been applied.

## 3 Results

### 3.1 ECAT

**Fig. 1:**
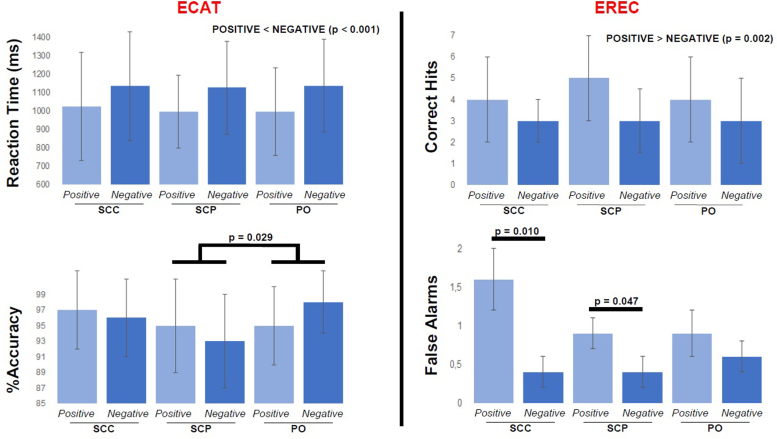
Emotionally-valenced verbal cognitive tests. (*ECAT, upper part*) Bar-chart presenting each treatment group’s mean reaction time (with the corresponding S.D.) for liking positive personality traits and disliking negative ones. Independent of the treatment group and on average, participants tend to respond faster in liking personality traits with a positive valence than in disliking personality traits with a negative valence (the mean difference is 128 ms with 95% CI 76–177 ms, p < 0.001). (*ECAT, lower part*) Bar-chart presenting each treatment group’s mean %accuracy score (with the corresponding S.D.) for correctly categorising the personality traits as likable and dislikable. The subcutaneous-pulsatile treatment group (SCP) shows a significantly smaller %accuracy compared to the oral treatment group (PO) (the mean difference is 2.9% with 95% CI 0.3–5.5%, p = 0.029), and is also smaller (though statistically non-significant) in relation to the subcutaneous-continuous treatment group (SCC). (*EREC, upper part*) Bar-chart presenting each treatment group’s mean number of personality traits correctly recalled by the subjects (with the corresponding S.D.). Independent of the treatment group and on average, participants tend to recall more positively valenced personality traits than negatively valenced. (*EREC, lower part*) During the emotional recall task, apart from any accurate recalls, the volunteers were mistakenly recording positive and negative personality traits that they thought they’ve encountered during the ECAT session (which took place on average 8.5 min earlier). Participants in the SCP and SCC have mistakenly recalled significantly more positive than negative personality descriptors [mean difference 0.5 with 95% CI 0–1, p = 0.047, and 1.3 with 95% CI 0.4–2.2, p = 0.010, respectively]. ECAT: emotionally-valenced, self-referral word categorisation task, EREC: ECAT-related free recall task, S.D.: standard deviation.

The main effect of treatment might interact with subjects’ performance [the F-test just misses significance, F(1.676, 20.113) = 3.622, p = 0.052, ω^2^ = 0.16]. Pairwise comparisons with Bonferroni adjustment revealed that, independent of word valence, participants undergoing subcutaneous-pulsatile treatment were less accurate at correctly classifying the personality traits compared to both other treatment groups (the mean difference with the subcutaneous-continuous treatment group is 2.7% with 95% CI − 0.7% to 6.1%, p = 0.141, and the mean difference with the oral treatment group is 2.9% with 95% CI 0.3–5.5%, p = 0.029). No differences were estimated between treatment groups in their reaction times, independent of valence. Across all groups, positively valenced personality traits were quicker classified as such compared to the negatively valenced ones (p < 0.001) (Fig. 1).

### 3.2 EREC

Analysis of variance elicited a two-way interaction of [valence] × [cortisol dynamics] on the number of personality traits falsely recalled by the participants (i.e. false alarms; traits that the subjects thought they’ve seen during the ECAT session, but were not actually presented) [F(1.853, 24.089) = 4.052, p = 0.033, ω^2^ = 0.17]. Pairwise comparisons with Bonferroni adjustment have been performed to investigate any significant interactions of the simple main effects of each the two factors on the number of these inaccurate recalls; under the subcutaneous-pulsatile or the subcutaneous-continuous treatment, when examining false alarms, participants mistakenly recall significantly more positive than negative personality descriptors (mean difference 0.5 with 95% CI 0–1, p = 0.047, and mean difference 1.3 with 95% CI 0.4–2.2, p = 0.010 respectively). No differences were estimated between the treatment groups in their capacity to recall personality traits that were actually presented (i.e. correct hits), independent of valence. Across all groups, subjects could recall more positively valenced personality traits compared to negatively valenced ones (p = 0.002) (Fig. 1).

### 3.3 EMA

**Fig. 2:**
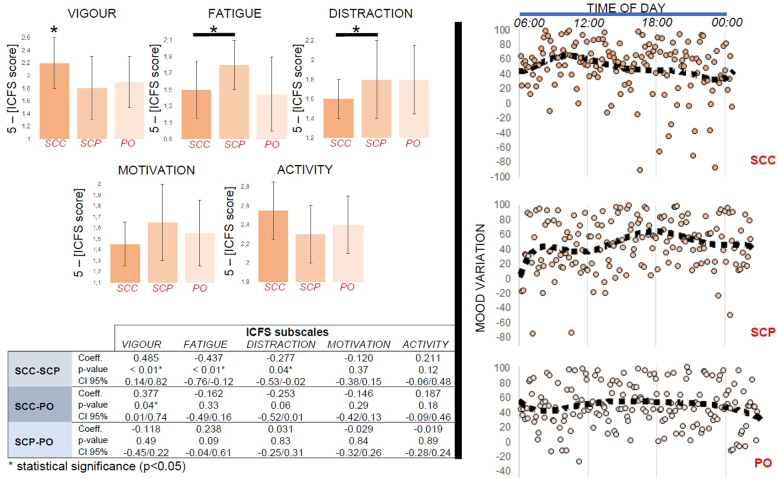
Self-perceived morning well-being and diurnal mood variation. (*Left panel*) Bar-charts showing each treatment group’s mean scores (with the corresponding S.D.) in the 5 subscales of the Identity-consequence fatigue scale (ICFS). The table underneath presents the p-values of the pairwise comparisons. Ratings of vigour where markedly higher in the subcutaneous-continuous treatment group (SCC) compared to the other modes of hydrocortisone replacement. Moreover, ratings of distraction were significantly lower (i.e. the ability to concentrate was higher) in the SCC compared to the subcutaneous-pulsatile treatment group (SCP). Similarly, with ratings of fatigue. There was no good evidence for an effect of treatment on ICFS ratings related to motivation or activity. To improve clarity, all values have been reversed [5-(ICFS score)]. (*Right panel*) The diurnal mood variation (100: maximally positive mood, − 100: maximally negative mood) was constructed by subtracting the negative mood ratings from the corresponding positive mood ratings (as estimated by the self-perceived reactivity and well-being items contained in the ecological momentary assessment, see Supplementary Materials and Methods) of days 3 and 4 of each subject and treatment arm. This approach has been suggested by prior research (Basabe et al., 2002). In SCC, mood changes are following the circadian glucocorticoid rhythm, possibly with a small delay. The best mood of the day occurs just before midday. On the contrary, when exposed to a tightly controlled, pulsatile (ultradian) glucocorticoid rhythm (SCP), the diurnal mood variation is dissociated from the hormone’s circadian profile and the best mood of the day occurs in the evening. Finally, when exposed to a non-physiological ultradian glucocorticoid rhythm, characterised by infrequent and inconsistent pulses and minimal glucocorticoid availability over the night sleep (PO), the diurnal mood variation is less pronounced, compared to the other modes of hydrocortisone bioavailability. CI: confidence intervals, PO: oral treatment group, S.D.: standard deviation.

Ratings of VIGOUR where markedly higher, and those of DISTRACTION significantly lower (i.e. the ability to concentrate was higher), during the subcutaneous-continuous treatment compared to the other modes of hydrocortisone replacement. On the other hand, ratings of FATIGUE were markedly higher during the subcutaneous-pulsatile treatment compared to the other modes of hydrocortisone replacement (Fig. 2).

Individual analysis of each treatment condition over the 24-h cycle revealed a circadian decrease of positive mood ratings during the subcutaneous-continuous treatment, a circadian increase in positive mood ratings during the subcutaneous-pulsatile treatment and no change of positive mood ratings during the oral treatment. The opposite pattern was found for negative mood rating; during the subcutaneous-continuous treatment negative mood ratings exhibited a circadian increase, during the subcutaneous-pulsatile treatment they showed a circadian decrease and during the oral treatment no change was found (Fig. 2, Table S2).

We also performed an individual analysis on the oscillatory activity of mood for each treatment condition across study days. For the subcutaneous-continuous treatment, no individual effect of day was found on negative or positive mood ratings. On the contrary, for the subcutaneous-pulsatile treatment, negative mood started decreasing on study day 3 and continued to do so on study day 4, while positive mood ratings increased on study day 4. For the oral treatment, there was some indication for the increase of negative mood ratings on study day 3 and a restoration on study day 4, while positive mood ratings were increased on study day 4 as well (Table S2).

### 3.4 Resting state functional neuroimaging data

**Fig. 3:**
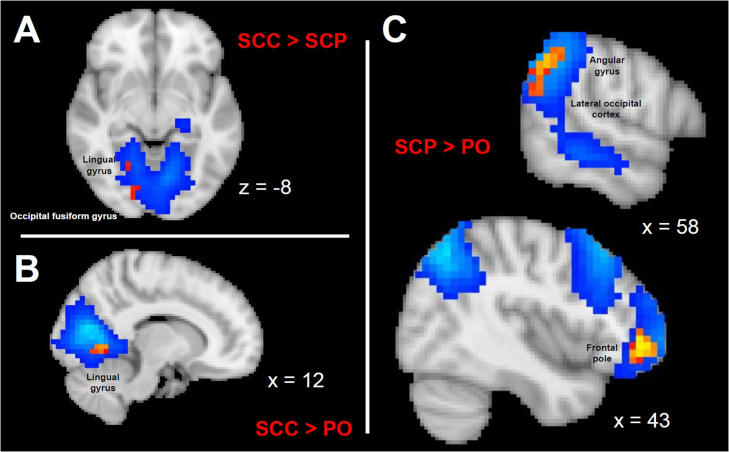
Functional connectivity within large-scale resting state networks. Aspects of the differential within-network connectivity of large-scale resting state brain networks in healthy volunteers exposed to different glucocorticoid ultradian rhythms, as highlighted by independent component analysis and dual regression in FSL (corrected for multiple comparisons at voxel level, threshold p < 0.05). The blue/light blue colormaps indicate the resting state networks, while the yellow-red colormaps those parts of the network with the altered, treatment-related functional activity. (A) Resting state network involving bilateral medial occipital cortices, previously described as a consistent resting state network in man, [see Chen et al. (2008)]. The right-sided lingual gyrus and occipital fusiform gyrus are differentially functionally connected with the rest of the network depending on whether subjects are exposed to subcutaneous pulsatile versus non-pulsatile glucocorticoid replacement therapy. The coordinate in the z-axis (axial view) refer to MNI152 standard space. (B) Resting state network involving bilateral medial occipital cortices, also described as a consistent resting state network in man [see Chen et al. (2008)]. The right-sided lingual gyrus is differentially functionally connected with the rest of the network depending on whether subjects are exposed to subcutaneous continuous versus oral glucocorticoid replacement therapy. The coordinate in the x-axis (sagittal view) refer to MNI152 standard space. (C) Resting state network involving the right-sided dorsolateral prefrontal and parietal cortices, also described as a consistent resting state network in man [see Chen et al. (2008)]. The angular gyrus, lateral occipital cortex and frontal pole are differentially functionally connected with the rest of the network depending on whether subjects are exposed to subcutaneous pulsatile versus oral glucocorticoid replacement therapy. The coordinate in the x-axis (sagittal view) refer to MNI152 standard space. FSL: FMRIB software library (Oxford University), PO: oral treatment group, SCC: subcutaneous-continuous treatment group, SCP: subcutaneous-pulsatile treatment group. (For interpretation of the references to colour in this figure legend, the reader is referred to the web version of this article.)

**Fig. 4:**
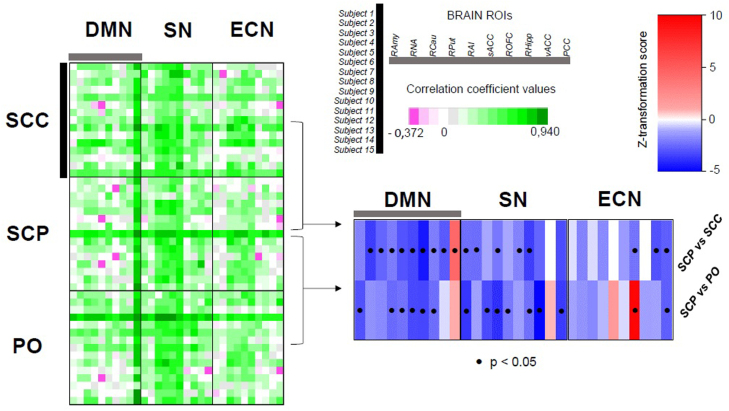
Functional connectivity of corticolimbic regions with the default mode, salience and executive control networks. (*Left part of the figure*) Green-purple colour heatmap of the correlation coefficient values (Spearman’s rank-order correlation test) between the 10 preselected ROIs and the main large-scale resting state brain networks (DMN, SN, ECN) per treatment group (SCC, SCP, PO) and subject. A mean value has been calculated from the resulting correlation coefficients per ROI and treatment group, and subsequently these mean values have been compared at treatment group-level after applying Fisher z-transformation (blue-red colourmap, significance threshold p < 0.05) (*lower right part of the figure*). As expected, based on prior knowledge, ROIs (belonging to the corticolimbic system of the brain) show higher functional connectivity to SN, and less to DMN (with the prominent exception of PCC) and ECN. Most ROIs are evenly or stronger connected with the three brain networks in the glucocorticoid treatment mode lacking the ultradian pulses (SCC) compared to the one comprising the approximation of the normal ultradian rhythm (SCP), with the exception of the PCC whose connectivity with DMN is stronger in SCP compared to SCC. Similarly, most ROIs are evenly or stronger connected with the three brain networks in the oral glucocorticoid treatment (PO) compared to the one comprising the approximation of the normal ultradian rhythm (SCP), with the exceptions of the functional connectivities of PCC with DMN, vACC with SN, and ROFC and RAI with ECN, which are is stronger in SCP compared to PO. DMN: default mode network, ECN: executive control network, PCC: posterior cingulate, RAI: right anterior insula, RAmy: right amygdala, RCau: right caudate, RHipp: right hippocampus, RNA: right nucleus accumbens, ROFC: right orbitofrontal cortex, ROIs: regions of interest, RPut: right putamen, sACC: middle part of the anterior cingulate, SN: salience network, vACC: ventral part of the anterior cingulate PO: oral treatment group, SCC: subcutaneous-continuous treatment group, SCP: subcutaneous-pulsatile treatment group. (For interpretation of the references to colour in this figure legend, the reader is referred to the web version of this article.)

**Fig. 5:**
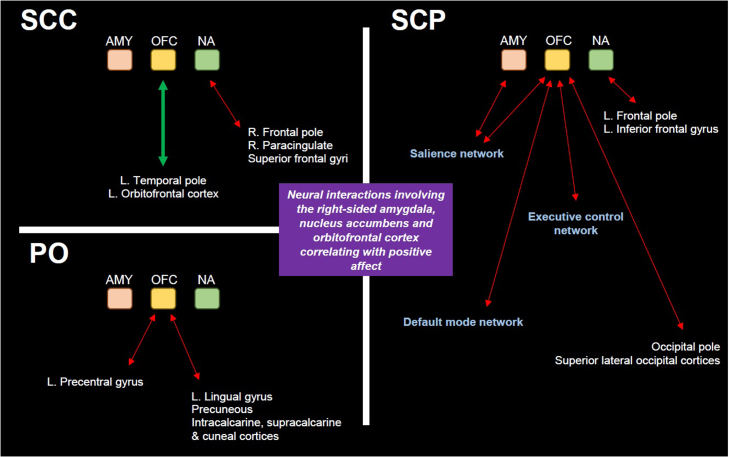
Associations between positive mood oscillations and amygdala-, nucleus accumbens- and orbitofrontal cortex-related brain network dynamics. Within-treatment-group correlation analysis between three seed-related resting state networks and the scores of a positive affect scale developed based on volunteers’ ratings in relevant questions (via ecological momentary assessment, see Supplementary Materials and Methods). For each treatment group, positive affect variation has been associated with a distinct pattern of interactions between the seed regions (right amygdala/ AMY, right lateral orbitofrontal cortex/ OFC and right nucleus accumbens/ NA) and other areas of the brain. In the subcutaneous-continuous treatment group (SCC), the across-individuals variation in positive affect can be explained by (i) the degree of attenuation in the functional connectivity between NA and parts of the right paracingulate cortex, superior frontal gyri and the right frontal pole (dotted red line), and (ii) the degree of increase in the functional connectivity (continuous thick green line) between OFC and parts of the left-sided orbitofrontal cortex and temporal pole. In the oral treatment group (PO), the across-individuals variation in positive affect can be explained by the degree of attenuation in the functional connectivity between the OFC and same-sided regions responsible for the processing of somatosensory input (lingual gyrus, cuneal cortex and areas around the calcarine sulcus) and the left precentral gyrus (dotted red lines). Finally, in the subcutaneous-pulsatile treatment group (SCP), the across-individuals variation in positive affect can be explained by the degree of attenuation in the functional connectivity (i) between AMY and parts of the right-sided salience network, (ii) between NA and left-sided frontal pole and inferior frontal gyrus, and (iii) between OFC and extensive brain regions, from both hemispheres, involving core nodes of the salience, default-mode and executive control networks, as well as other regions responsible for visual processing (dotted red lines). L.: left, R.: right. (For interpretation of the references to colour in this figure legend, the reader is referred to the web version of this article.)

The ICA approach revealed various large-scale RSNs which possess a differential within-network connectivity depending on the underlying mode of hydrocortisone administration (Fig. 3) (Menon, 2011, Chen et al., 2008). Moreover, the seed-based functional connectivity analysis indicates that highly glucocorticoid-sensitive, corticolimbic regions (like the amygdala, the dorsal and ventral striatum, cingulate, hippocampus, orbitofrontal cortex and insula) show a differential connectivity with the DMN, SN and ECN depending on the underlying glucocorticoid rhythm (Fig. 4). Nevertheless, these hormonal rhythm-dependent changes do not alter the characteristic pattern of dynamics between some of the ROIs and the three large-scale resting state networks, based on prior knowledge (Fig. S6). For instance, the functional connectivity of posterior cingulate with the DMN is stronger in the SCP treatment group compared to the other modes of hydrocortisone replacement, although in all three treatment groups the BOLD signal of posterior cingulate is, as expected, highly correlated with the DMN. Or the functional connectivity of the anterior insula with the SN is weaker in the SCP treatment group compared to the other modes of hydrocortisone replacement, although in all three treatment groups the BOLD signal of anterior insula is, as expected, highly correlated with the SN. Finally, distinct patterns of neural network dynamics involving the right amygdala, the nucleus accumbens and especially the orbitofrontal cortex correlate with positive affect variation for each mode of hydrocortisone replacement (Fig. 5 and Table S4).

## 4 Discussion

In this study, we performed a block and replace protocol (combining a glucocorticoid synthesis-blocking agent with three different modes of hydrocortisone replacement) together with computerised cognitive testing, ecological momentary assessment and resting state functional imaging of the brain of healthy individuals to provide evidence on the possible significance of glucocorticoid rhythmicity in mood regulation and neural dynamics. Our data suggest that glucocorticoid pulsatility constitutes a regulatory factor for processes involved in cognitive psychophysiology, including (i) self-perceived domains of well-being, (ii) daily mood oscillations, and (iii) resting state neural dynamics, independently and in the context of mood regulation.

The use of VASQ for assessing mood has been long proposed as a useful clinical tool, and its reliability and validity verified (Folstein and Luria, 1973). This approach has proved more reliable in data collection, compared to the traditional pen and paper method (Whybrow et al., 2006), especially in the context of ecological momentary assessment studies, where participants require to record data multiple times daily, while being engaged with their non-controlled, often outdoor, routine activities (Shiffman et al., 2020). Our version of VASQ has an established record of applicability in monitoring positive and negative affect oscillations for investigating behaviour in substance dependence studies (Ferguson and Shiffman, 2011, Shiffman et al., 2013, Peacock et al., 2015).

Different temporal patterns of hydrocortisone replacement alter the morning (post-wakening) levels of self-perceived fatigue, vigour and concentration. At this time subjects undergoing the non-pulsatile treatment subjectively feel more vigorous and able to concentrate compared to pulsatile hormonal replacement whereas subjects on the pulsatile treatment feel more fatigued (Fig. 2). On the other hand, subjects undergoing non-pulsatile treatment subsequently showed a decrease in positive mood and an increase in negative mood throughout the rest of the day whereas pulsatile treatment resulted in an increase in positive mood and a decrease in negative mood which was maintained for the rest of the day (Table S2). These data suggest that in our protocol the pulsatile infusions resulted in less liveliness in the morning but provided sustained positive mood for longer periods during the day. This may well relate to the temporal mode of the hydrocortisone delivery. In the non-pulsatile infusion, subjects have a large pulse of cortisol in the early morning but have no further pulses during the day. During the pulsatile infusion, subjects have a smaller pulse in the early morning but continue to experience peaks and troughs of cortisol throughout the day.

Trying to place these data into the psychophysiological context, it helps to briefly summarise current evidence related to mood regulation under normal conditions. In healthy subjects, it has been shown that mood exhibits a circadian variation (Porto et al., 2006), driven mainly by the positive mood. The best mood of the day (greatest difference between positive and negative mood) takes place at around midday, when positive mood reaches its maximum. After that, it drops steadily reaching its minimum at around midnight (Murray et al., 2002). Furthermore, behaviour associated with positive mood (laughing, socialising, singing) is also characterised by a circadian variation, starting from low levels after the morning awaking, and reaching its maximum 5–12 h later (Hasler et al., 2008). In addition, features like fatigue, confusion and total mood disturbance are high by the morning awaking and start decreasing as the day progresses, contrary to vigour which starts from lower levels by the morning awaking and increases during the day (Monk et al., 1997, Kline et al., 2010). Thus, it seems that daily mood variation in subjects undergoing the pulsatile replacement (which most closely approximates to the physiological ultradian cortisol rhythm) lies closer to current observations of the normal state. This notion is further supported by the results of a psycholinguistic study of millions of twitter posts recording the use of words that relate to mood and thus obtain objective indices of population based circadian mood oscillations (Dzogang et al., 2017). In that study, two maxima were observed during the weekday: one in the morning and one in the evening/ night, like the pattern of daily mood oscillations in our pulsatile treatment group. Although these are just associations, these all point to the importance of combining cutting edge technologies with dynamic neurobehavioural tools, to establish the mechanisms underlying the relationship between cortisol rhythms and mood oscillations.

In our current study of brain connectivity we also find that different patterns of hydrocortisone replacement are accompanied by changes in the within-network functional connectivity of various large scale RSNs, and the functional connectivity of the three main RSNs of the human brain (DMN, SN, ECN) with corticolimbic regions. Within the network responsible for processing visually relevant information, the right lingual gyrus showed stronger functionally connectivity with the occipital cortices bilaterally when subjects receive non-pulsatile glucocorticoid replacement compared to pulsatile treatment (either optimal or non-optimal) (Fig. 3), while the right-sided orbitofrontal cortex showed weaker functional connectivity with the default mode and the salience networks when subjects received the pulsatile subcutaneous treatment compared to the other modes of hydrocortisone replacement (Fig. 4). Furthermore, positive mood variations were associated with different patterns of amygdala-, nucleus accumbens- and orbitofrontal cortex-related brain network dynamics depending on the mode of hydrocortisone replacement (Fig. 5). Finally, changes in accuracy of classification and quality of recollection of emotionally-valenced, self-referral descriptors were observed between different treatment groups.

Mood regulation encompasses the interaction between large-scale resting state networks of the brain (responding to internal or external cues that modulate the level of arousal, saliency detection and self-awareness) with corticolimbic regions (responsible for further integrating emotional cues and stimuli differentially mobilising the stress and/or reward system). For instance, it has been shown that negative mood is correlated with increased within-network connectivity of the SN and decreased connectivity of the DMN (Harrison et al., 2008, Provenzano et al., 2019), while engaging with aesthetically pleasing cues increases the functional connectivity of DMN (Vessel et al., 2012). Moreover, orbitofrontal cortex is a crucial node for positive mood regulation (Nitschke et al., 2004, Rao et al., 2018). In our study, pulsatile treatment increases the within-network connectivity of DMN and reduces that of SN (Fig. 4) compared to the other, suboptimal modes of hydrocortisone replacement, a pattern of large-scale neural dynamics facilitating positive mood regulation in keeping with these previous studies. Furthermore, pulsatile treatment dissociates the functional interplay of the right orbitofrontal cortex with DMN and SN (Fig. 4), a condition which also correlates with positive mood regulation in our study (Fig. 5). Collectively, our findings indicate that changes in the ultradian dimension of cortisol dynamics affect cognitive psychophysiology under non-stressful conditions by modulating the neural dynamics of resting state networks of the human brain.

Our findings have major clinical implications for efforts to optimise glucocorticoid replacement therapies. Current protocols recommend the oral administration of hydrocortisone 2–3 times daily, with the morning dose being at least 50% of the total daily amount. We’ve integrated this treatment strategy into our study, as one of the three different modes of cortisol replacement. This strategy creates a form of suboptimal hormonal pulsatility, with the circadian peak occurring after waking up in the morning, with a much smaller number of daily pulses, and with an inconsistent, usually much-longer-than-normal, duration of inter-pulse intervals. In addition to our previously published report on emotional processing (Kalafatakis et al., 2018), the data we present here further indicate that this commonly used replacement protocol results in significant neuropsychological differences to a more physiological regime.

Over the last decade, an alternative approach for cortisol replacement has been proposed, involving the continuous, non-pulsatile pump-mediated subcutaneous infusion of hydrocortisone, mimicking the circadian but ignoring the ultradian pattern of plasma cortisol. We felt it was important to integrate this strategy into our study as there is evidence it might improve markers of fatigue, vitality and physical function, especially after a period of many weeks to months (Oksnes et al., 2014, Gagliardi et al., 2014, Nella et al., 2016, Mallappa et al., 2018). Although our study was able to replicate some of these findings over a much shorter treatment period, in our healthy subjects, we were also able to show marked differences between the pulsatile and non-pulsatile modes of replacement on the mood response of our subjects over the day. That type of knowledge will be crucial as we develop a personalised medicine approach to glucocorticoid hormone replacement.

Due to the inter-individual differences in ultradian patterns of cortisol secretion it would not have been feasible to include a non-treated ‘control’ group in this study. We would simply not have known the timing of the endogenous rhythm in any subject in relation to our outcome measures; the computerised psychological tests or the fMRI scanning protocol would have been performed at random times during the subjects’ ultradian cycles, preventing the ability to place the results into a biological context and interpret them in relation to physiology. In the future, using smart devices able to perform continuous, minimally invasive estimations of plasma cortisol dynamics or mathematical models able to predict daily plasma cortisol dynamics from only a few blood sampling measurements, it might be possible to reliably include such a “control” group into studies of similar design.

A limitation of this study was the relatively short duration over which we could ethically maintain our volunteers on the block and replace treatment regimens. A previously published trial however has already validated and standardised our block and replace protocol and showed biochemical evidence of successful recapitulation of physiological levels of plasma cortisol by all replacement strategies (Kalafatakis et al., 2016b). Future studies in patients with adrenocortical insufficiency are now needed with much longer duration in each treatment group to better characterise the neuropsychological effects of our novel, pump-mediated, pulsatile subcutaneous replacement therapy. This should hopefully provide the evidence needed to improve glucocorticoid treatment regimens, help reduce the morbidity of current replacement therapy and reduce the long-term neuropsychiatric problems of patients with adrenocortical insufficiency.

## Funding and disclosure

Funding for this work was provided by the Medical Research Council [DCS Grant MR/J0125481/1] and the Wellcome Trust [Grant 089647/Z/09/Z]. CJH was supported by the Oxford Health NIHR Biomedical Research Centre. All authors would like to declare no competing financial interests in relation to the work described.

## CRediT authorship contribution statement

KK conducted the study, analysed the data and prepared the manuscript. GMR, SLL, MNM, CLH designed the study, supervised data analysis and significantly contributed to paper writing. MG, SGF designed the ecological momentary assessment-related parts of the study, co-analysed the relevant data and added the corresponding parts to the manuscript. NM, AW co-conducted the study and assisted in paper writing. JT, PM analysed parts of the neuroimaging data and assisted in paper writing. JCB, NJT advised on study design, neuroimaging data analysis and assisted in paper writing.

## Footnotes

Appendix A Supplementary data associated with this article can be found in the online version at doi:10.1016/j.psyneuen.2020.105096.
