# Supplementary material for: Glucocorticoid ultradian rhythmicity differentially regulates mood and resting state networks in the human brain: A randomised controlled clinical trial
Source: Psychoneuroendocrinology. 2021 Feb;124:105096. doi: 10.1016/j.psyneuen.2020.105096 (PMC7895801; doi:10.1016/j.psyneuen.2020.105096)
Supplement: Supplementary file 1 — Supplementary material [file mmc1.pdf]

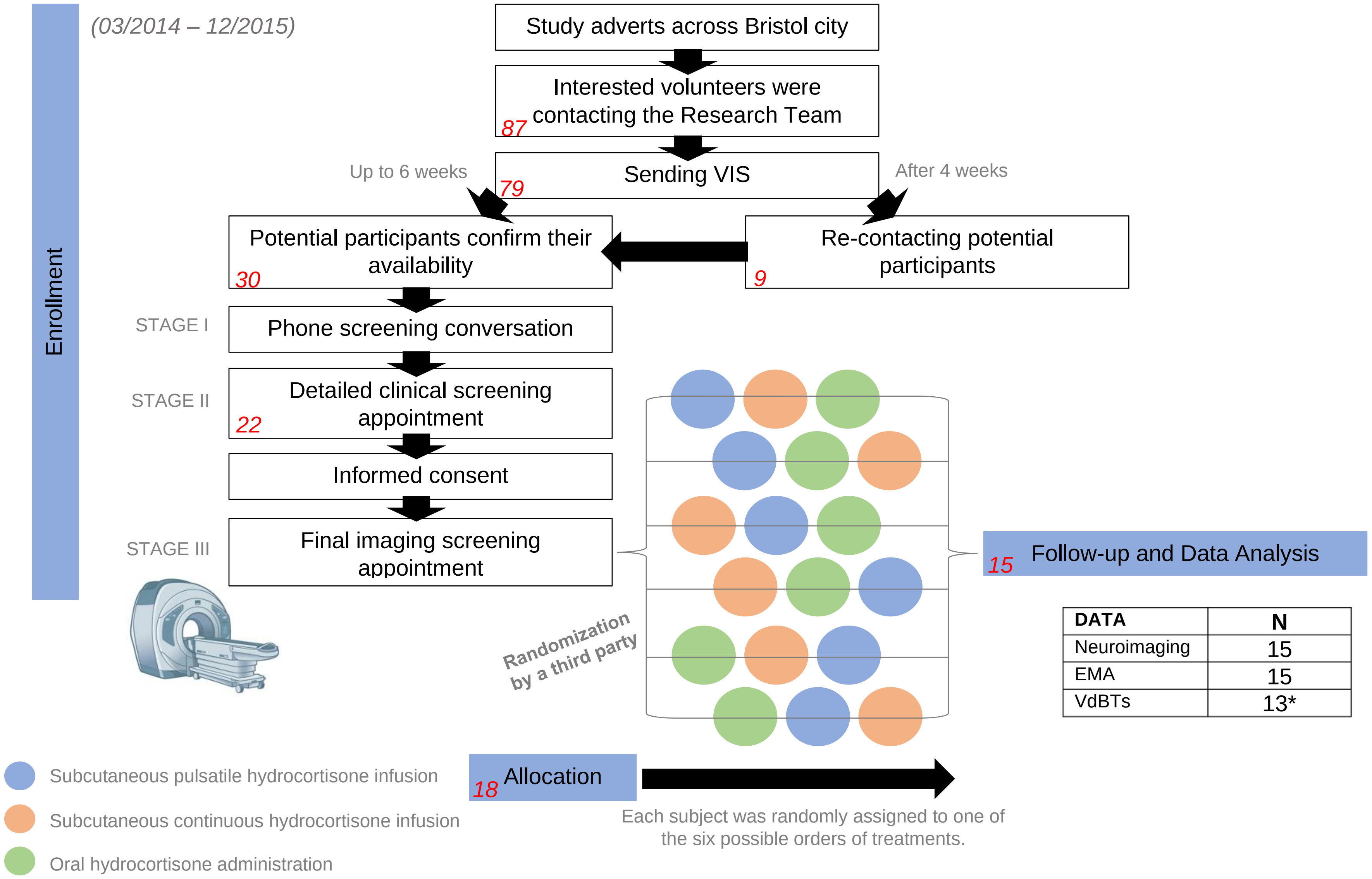

DATA	N
Neuroimaging	15
EMA	15
VdBTs	13*

Figure S1

Flow diagram based on CONSORT guidelines. Enrollment (took place between March 2014 and December 2015); after sending the volunteer information sheets (VIS) via e-mail to interested subjects, participants were given 6 weeks to reply. The study researchers were optionally sending a reminder to interested individuals if they hadn't reply for at least 4 weeks. For the clinical trial, 87 subjects have overall expressed their interest. Eight of them were immediately excluded from the study being either female subjects or for medical reasons. The corresponding VIS was sent to the rest of them. Nevertheless, only 30 eventually replied, re-confirming their interest and explicitly stating their availability to undergo the three stages of the screening process. Seven subjects were excluded during the phone screening conversation based on the exclusion criteria [see Kalafatakis et al. (2016b)], while another subject, although passed the phone screening, lost his interest for the study. Twenty-two healthy male volunteers participated successfully in the detailed clinical screening appointment and gave an informed consent, although 4 of them lost their interest for the study prior coming to the last part of the screening process (which ensured that subjects were eligible to be scanned, and included the acquisition of high-resolution, anatomical MR images). The remaining 18 subjects were randomly allocated (by a third party to ensure the double-blind nature of the study) to one of the six possible orders of treatments, with the limitation that that the difference in the final number of subjects between any order of treatments should not exceed 1, when the total number of participants reaches fifteen. Three more subjects were excluded from the study, after they've been recruited and randomized because they were positive in the test for drugs of abuse just before starting their first study arm. Eventually, each subject underwent all 3 treatment arms (three-way crossover study). In each treatment arm, subjects were required to take the same daily regimen of tablets and remain connected to a subcutaneous infusion pump (placebo-controlled, double-blind study). There was a minimum period of 2 weeks between treatment arms. Fifteen subjects completed the study, were followed-up (the next day after finishing each study arm) and their data analysed. Two subjects were systematically performing poorly in the verbal-dependent behavioural tests (VdBTs). Both participants were non-native English speakers (coming from Latin America and Africa respectively); the low scores reflect their occasional limited understanding of the verbal components of the tests, for which they've chosen to respond randomly. This could introduce a systematic bias; therefore, both subjects were excluded from data analysis (*). EMA: ecological momentary assessment

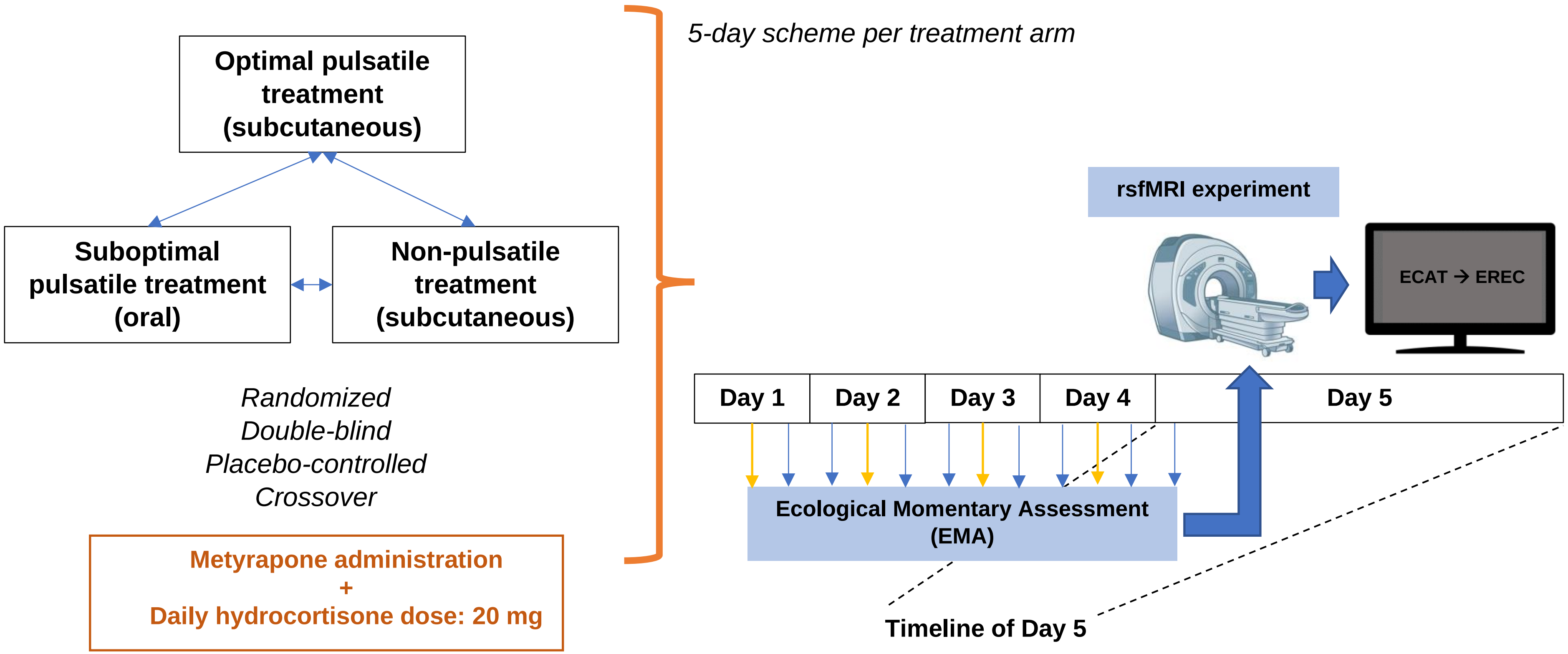

Timeline of Day 5

	EMA morning report	rsfMRI session	ECAT	EREC
**SCC**	07:27 +/- 00:23 am	9:00 +/- 00:09 am	10:33 +/- 00:09 am	10:46 +/- 00:09 am
**SCP**	07:40 +/- 00:33 am	9:02 +/- 00:14 am	10:34 +/- 00:07 am	10:47 +/- 00:07 am
**PO**	07:33 +/- 00:25 am	9:03 +/- 00:05 am	10:28 +/- 00:04 am	10:41 +/- 00:04 am

Figure S2

Overview of the type and timeline of the clinical trial. Fifteen subjects have participated in a randomised, double-blind, placebo-controlled crossover study. Each of them underwent three 5-day long periods of hydrocortisone replacement therapy [after concurrent pharmacological blockage of endogenous cortisol biosynthesis via the oral administration of metyrapone, for more details see Kalafatakis et al. (2016b)] with a minimum interval between the treatment periods of 2 weeks. For each treatment period hydrocortisone was substituted in one of the following modes; either subcutaneously, via a pump, delivering pulses of hydrocortisone every 3 hours, with the pulse amplitude varying depending on the time of day (big pulses of 4 mg of hydrocortisone were being infused at 03:00 am, 06:00 am and 09:00 am, intermediate pulses of 2.3 mg of the hormone were being infused at 12:00 pm, 03:00 pm and 06:00 pm, and small pulses of 0.5 mg of the hormone were being infused at 09:00 pm and 12:00 am) (SCP). Or subcutaneously, via a pump, infusing hydrocortisone continuously in a rate-varying manner depending on the time of day (starting at 2 mg/hour at 02:00 am, dropping to 1 mg/hour at 08:00 am, further dropping to 0.4 mg/hour at midday, and again to 0.1 mg/hour at 08:00 pm, before increasing back to 2 mg/hour at 02:00 am of the next day) (SCC). Or orally, three times a day (10 mg at breakfast, 5 mg at lunch and 5 mg at dinner) (PO). In all treatment modes the daily hydrocortisone dose was the same (20 mg). Each participant was allocated in a random order of treatment periods, and this order was unknown to him and the group of researchers; for every treatment period participants were given oral pills (hydrocortisone/placebo) with instructions on when to take them, and connected to a pump of subcutaneous drug delivery (placebo/hydrocortisone).

Starting after midday on day 1 of each treatment period, subjects were participating in an ecological momentary assessment (EMA) experiment mediated by an android phone they were constantly carrying with them. Through the android phone subjects were answering questions on their mood and reactivity either during fixed timepoints per day (in the morning after waking up, i.e. morning report, and in the evening after 19:00, i.e. evening report, blue arrows) or at random timepoints and for a random number of times throughout each day (yellow arrows) (for more details see supplementary figure 2). The EMA experiment was being completed after the subjects answered the questions of the morning report on the fifth study day. Thereafter, subjects were undergoing the rsfMRI experiment, followed by the ECAT and EREC.

ECAT: emotionally-valenced, self-referral word categorisation task, EREC: ECAT-related free recall task, rsfMRI: resting state functional magnetic resonance imaging

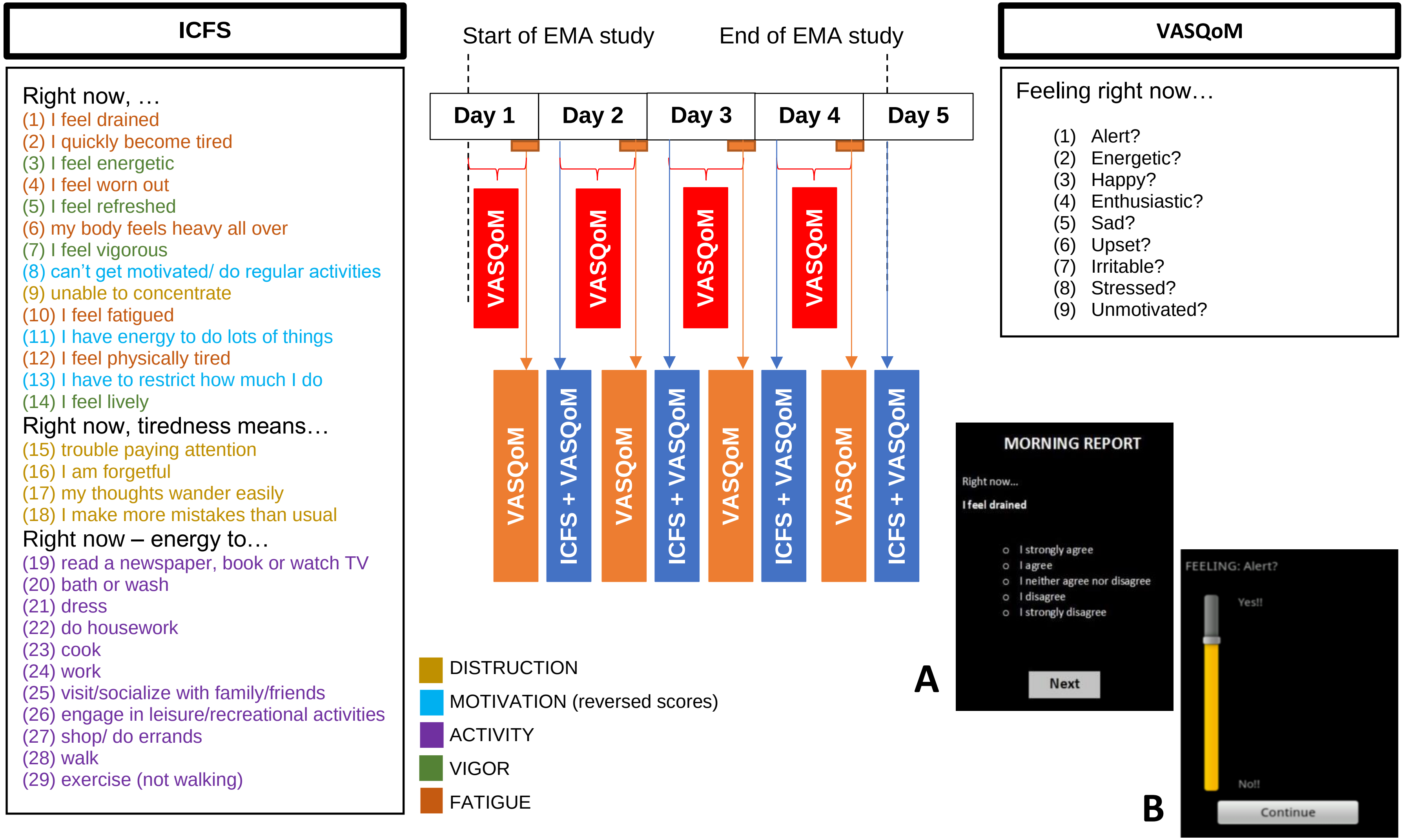
ICFS
Right now, …
(1) I feel drained
(2) I quickly become tired
(3) I feel energetic
(4) I feel worn out
(5) I feel refreshed
(6) my body feels heavy all over
(7) I feel vigorous
(8) can't get motivated/ do regular activities
(9) unable to concentrate
(10) I feel fatigued
(11) I have energy to do lots of things
(12) I feel physically tired
(13) I have to restrict how much I do
(14) I feel lively
Right now, tiredness means…
(15) trouble paying attention
(16) I am forgetful
(17) my thoughts wander easily
(18) I make more mistakes than usual
Right now – energy to…
(19) read a newspaper, book or watch TV
(20) bath or wash
(21) dress
(22) do housework
(23) cook
(24) work
(25) visit/socialize with family/friends
(26) engage in leisure/recreational activities
(27) shop/ do errands
(28) walk
(29) exercise (not walking)
Start of EMA study
End of EMA study
Day 1
Day 2
Day 3
Day 4
Day 5
VASQoM
VASQoM
VASQoM
VASQoM
VASQoM
ICFS + VASQoM
VASQoM
ICFS + VASQoM
VASQoM
ICFS + VASQoM
VASQoM
ICFS + VASQoM
DISTRUCTION
MOTIVATION (reversed scores)
ACTIVITY
VIGOR
FATIGUE
VASQoM
Feeling right now…
(1) Alert?
(2) Energetic?
(3) Happy?
(4) Enthusiastic?
(5) Sad?
(6) Upset?
(7) Irritable?
(8) Stressed?
(9) Unmotivated?
A
MORNING REPORT
Right now...
I feel drained
I strongly agree
I agree
I neither agree nor disagree
I disagree
I strongly disagree
Next
B
FEELING: Alert?
Yes!!
No!!
Continue

Figure S3

Overview of the ecological momentary assessment (EMA) study. The questionnaires delivered to the subjects via android phones contained two types of questions; (ICFS) 29 statements from the multi-dimensional identity-consequence fatigue scale, and (VASQoM) 9 questions on their self-perceived emotional state. Typically, five components were being constructed by the 29 statements of each ICFS, and the value of each component was being derived by the mean value from the answers of the participants in the corresponding statements (VIGOR was being constructed by answers in the statements 3/5/7/14, DISTRACTION by answers in the statements 9/15/16/17/18, FATIGUE by answers in the statements 1/2/4/6/10/12, MOTIVATION by answers in the statements 8/11/13, and ACTIVITY by answers in the statements 19-29). Subjects had to complete a morning report (after waking up), containing both the ICFS and VASQoM questionnaires (blue boxes), as well as an evening report, which was available from 19:00 until midnight and containing only the VASQoM questions (orange boxes). In between, subjects had to complete at random timepoints a random number of VASQoM questionnaires (red boxes). The EMA was starting around midday of the first treatment day (per study arm) and finishing after completing the morning report of the fifth treatment day (per study arm).

(A) An example of how each statement of the ICFS was displayed through the android phone to participants. The latter needed to choose the most appropriate answer from the following five: (value=1) I strongly agree, (value=2) I agree, (value=3) I neither agree nor disagree, (value=4) I disagree, (value=5) I strongly disagree.

(B) An example of how each statement of the VASQoM was displayed through the android phone to participants. The latter needed to move the slider up or down, to the appropriate level (in a 0-100 scale, with 0 being the absolute negative response and 100 the absolute positive).

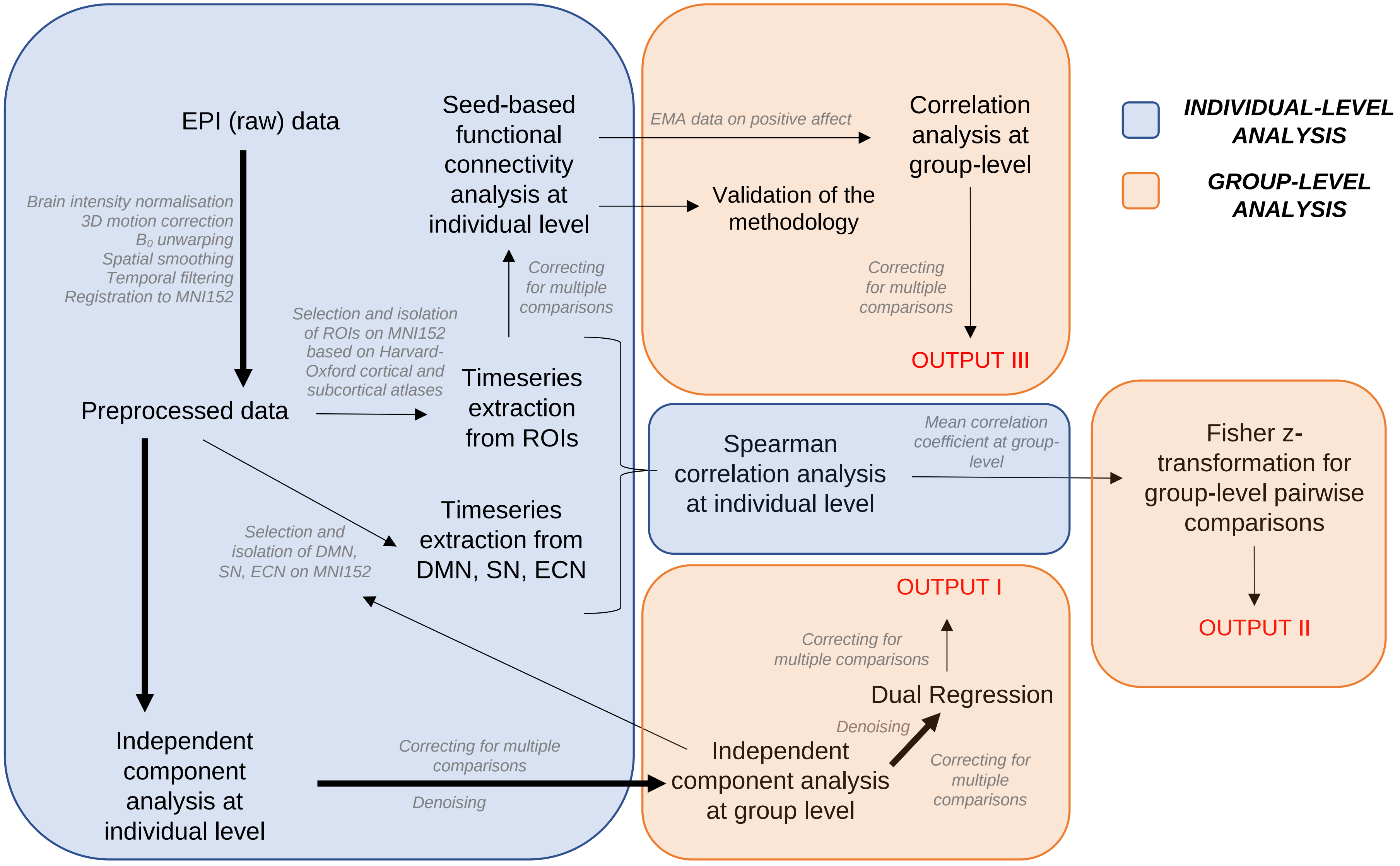
EPI (raw) data
Brain intensity normalisation
3D motion correction
B_0 unwarping
Spatial smoothing
Temporal filtering
Registration to MNI152
Preprocessed data
Seed-based functional connectivity analysis at individual level
Correcting for multiple comparisons
Selection and isolation of ROIs on MNI152 based on Harvard-Oxford cortical and subcortical atlases
Timeseries extraction from ROIs
Selection and isolation of DMN, SN, ECN on MNI152
Timeseries extraction from DMN, SN, ECN
Independent component analysis at individual level
Correcting for multiple comparisons
Denoising
EMA data on positive affect
Correlation analysis at group-level
Validation of the methodology
Correcting for multiple comparisons
OUTPUT III
Spearman correlation analysis at individual level
Mean correlation coefficient at group-level
Fisher z-transformation for group-level pairwise comparisons
OUTPUT II
OUTPUT I
Correcting for multiple comparisons
Dual Regression
Denoising
Independent component analysis at group level
Correcting for multiple comparisons
INDIVIDUAL-LEVEL ANALYSIS
GROUP-LEVEL ANALYSIS

Figure S4

Pipeline of the image processing and statistical analysis of the resting state functional neuroimaging data. Additional details can be also found in supplementary Figure 5.

DMN: default mode network, ECN: executive control network, EMA: ecological momentary assessment, EPI: echo planar imaging, MNI152: standard brain (by the Montreal Neurological Institute), ROIs: regions of interest, SN: salience network

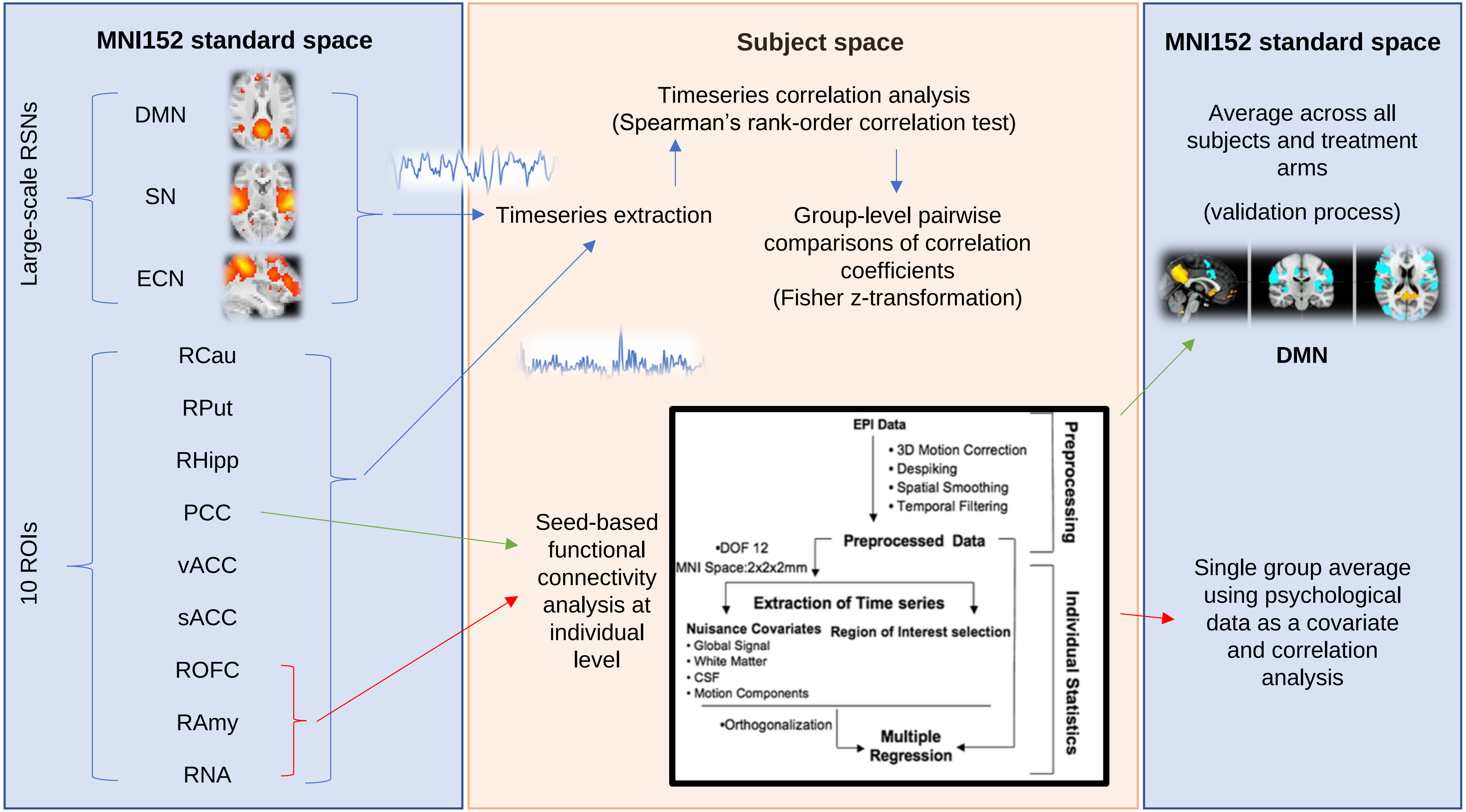
MNI152 standard space
Large-scale RSNs
DMN
SN
ECN
10 ROIs
RCau
RPut
RHipp
PCC
vACC
sACC
ROFC
RAmy
RNA
Subject space
Timeseries correlation analysis
(Spearman's rank-order correlation test)
Timeseries extraction
Group-level pairwise comparisons of correlation coefficients
(Fisher z-transformation)
Seed-based functional connectivity analysis at individual level
EPI Data
• 3D Motion Correction
• Despiking
• Spatial Smoothing
• Temporal Filtering
Preprocessing
•DOF 12
MNI Space:2x2x2mm
Preprocessed Data
Extraction of Time series
Nuisance Covariates
• Global Signal
• White Matter
• CSF
• Motion Components
Region of Interest selection
•Orthogonalization
Multiple Regression
Individual Statistics
MNI152 standard space
Average across all subjects and treatment arms
(validation process)
DMN
Single group average using psychological data as a covariate and correlation analysis

Figure S5

Additional information on the processing pipeline followed for performing a ROI-dependent analysis of the resting state functional neuroimaging data. Two independent approaches have been used; in the first of them (blue arrows) the timeseries of each of the 10 preselected ROIs have been correlated with the timeseries of each of the 3 main RSNs corresponding to each subject and treatment mode. A mean value has been calculated from the resulting correlation coefficients per ROI and treatment group, and subsequently these mean values have been compared at treatment group-level by applying Fisher z-transformation. The 3 main RSNs have been isolated from the group-level ICA of the resting state functional neuroimaging data across all subject and treatment group sessions. The second approach (red and green arrows) consisted of a validation step, as well as the main analysis step. We implemented the seed-based functional connectivity analysis proposed by Di Martino et al. (2008), and validated that it created sensible outputs when applied to our dataset; indeed, when using PCC as a seed for that type of analysis, and averaging the output across all subjects and sessions, we were able to show that PCC, precisely as expected from the literature [9], was positively correlating with brain regions like precuneous, medial prefrontal cortex and vACC (corresponding to DMN) and negatively correlating with brain regions like insular cortices, sACC, frontal and parietal regions (corresponding to the salience and executive control networks). Finally, we used RAmy, RNA and ROFC to perform a seed-based functional connectivity analysis at individual level, and subsequently perform an analysis of covariance at each treatment group-level between the seed-related networks and the corresponding degree of positive affect of the participants (as captured by the ecological momentary assessment morning report a few minutes prior the resting state neuroimaging experiment).

CSF: cerebrospinal fluid, DMN: default mode network, DOF: degrees of freedom, ECN: executive control network, EMA: ecological momentary assessment, EPI: echo planar imaging, ICA: independent component analysis, MNI152: standard brain (by the Montreal Neurological Institute), PCC: posterior cingulate, RAI: right anterior insula, RAmy: right amygdala, RCau: right caudate, RHipp: right hippocampus, RNA: right nucleus accumbens, ROFC: right orbitofrontal cortex, ROIs: regions of interest, RPut: right putamen, RSNs: resting state networks, sACC: middle part of the anterior cingulate, SN: salience network, vACC: ventral part of the anterior cingulate

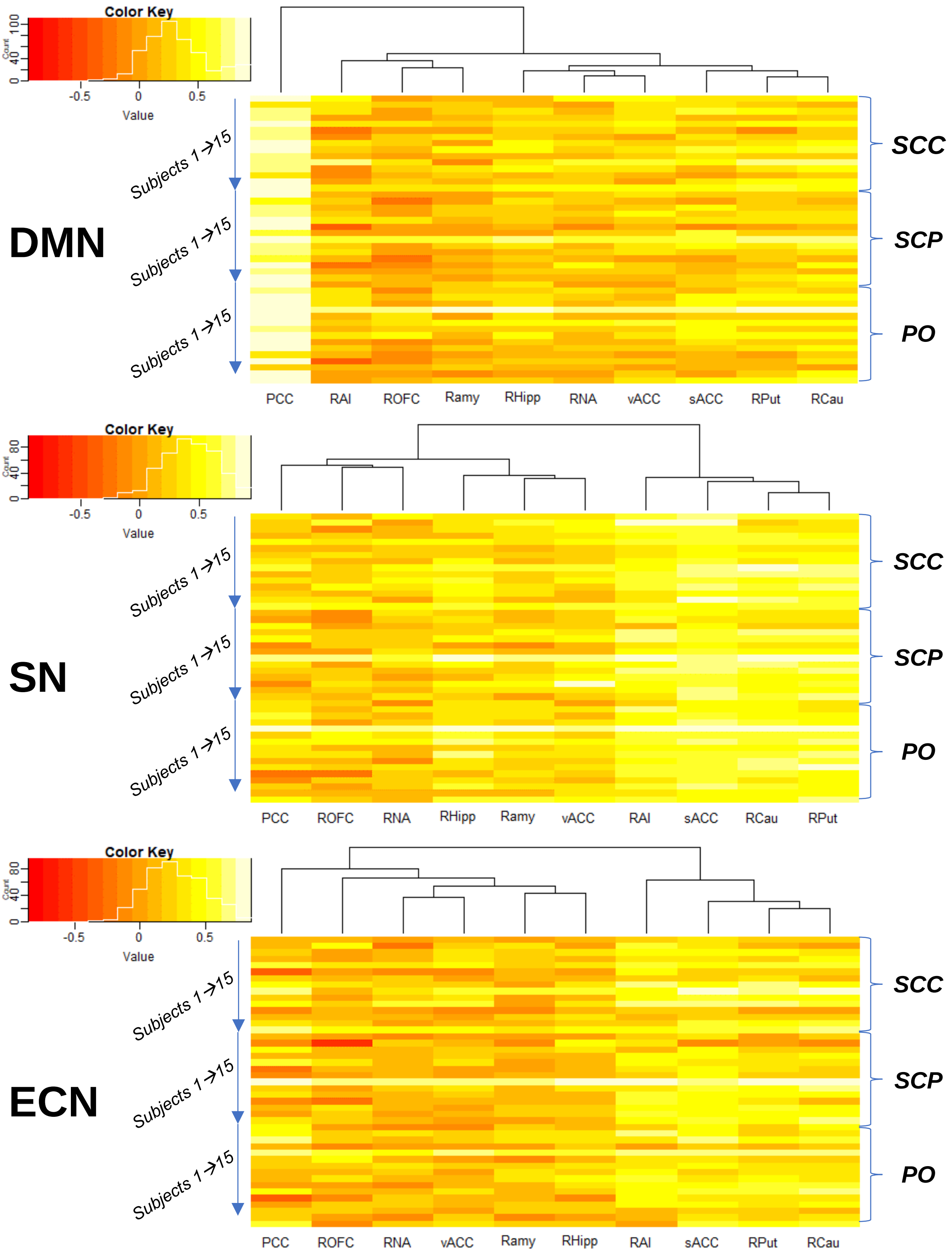

Color Key
Count
Value
DMN
Subjects 1→15
Subjects 1→15
Subjects 1→15
SCC
SCP
PO
PCC
RAI
ROFC
Ramy
RHipp
RNA
vACC
sACC
RPut
RCau
Color Key
Count
Value
SN
Subjects 1→15
Subjects 1→15
Subjects 1→15
SCC
SCP
PO
PCC
ROFC
RNA
RHipp
Ramy
vACC
RAI
sACC
RCau
RPut
Color Key
Count
Value
ECN
Subjects 1→15
Subjects 1→15
Subjects 1→15
SCC
SCP
PO
PCC
ROFC
RNA
vACC
Ramy
RHipp
RAI
sACC
RPut
RCau

Figure S6

A different perspective on presenting the correlation coefficient values between the timeseries of the three main large-scale resting state networks of the human brain (default-mode network/ DMN, salience network/ SN and executive control network/ ECN) and the preselected regions of interest (ROIs, see Supplementary Table 3). The heatmaps have been created in R; the treegrams show how close the different ROIs lie to each other based on the subject-wide pattern of their correlation coefficient values with the corresponding brain network. As expected, PCC area is highly correlated (across subjects and treatments) with the DMN, contrary to RAI, ROFC and RAmy, which show a reverse pattern of correlations. As also expected, RAI and sACC along with dorsal striatal regions (RCau and RPut) are highly correlated (across subjects and treatments) with SN, contrary to PCC. A similar pattern of correlations exists between the ROIs and ECN.

PCC: posterior cingulate, RAI: right anterior insula, RAmy: right amygdala, RCau: right caudate, RHipp: right hippocampus, RNA: right nucleus accumbens, ROFC: right orbitofrontal cortex, RPut: right putamen, sACC: middle part of the anterior cingulate, vACC: ventral part of the anterior cingulate

PARTICIPANTS	AGE	ETHNICITY	DEGREE OF RIGHT-HANDEDNESS	SMOKING HABBITS	ALCOHOL CONSUMPTION HABBITS	CAFFEINE CONSUMPTION HABBITS	WEIGHT	HEIGHT	BMI
1	33	Greece	+1.0	0	0	0	65	165	23.9
2	29	Nigeria	+1.0	0	0	0	68	177	21.7
3	25	Canada	+0.7	0	0	0	87	183	26.0
4	26	United Kingdom	+0.9	0	0	0	105	183	31.4
5	28	India	+0.4	0	3	0	88	187	25.2
6	22	United Kingdom	+0.9	0	10	1	67	184	19.8
7	30	United Kingdom	+0.6	0	3	2	86	189	24.1
8	31	Chile	+0.8	0	8	2	73	177	23.3
9	29	United Kingdom	+0.9	0	0	0	81	192	22.0
10	25	United Kingdom	+0.7	0	14	1	100	188	28.3
11	32	United Kingdom	+0.4	0	4	1	83	183	24.8
12	20	United Kingdom	+0.5	0	16	2	76	190	21.1
13	23	United Kingdom	+0.6	0	10	0	77	185	22.5
14	21	United Kingdom	+0.7	0	9	3	81	176	26.1
15	21	Australia	+0.3	0	10	2	66	179	20.6
**MEAN**	**26.3**		**+0.7**		**6**	**1**	**80.2**	**182.5**	**24.1**
**SD**	**4.2**		**0.2**		**5**	**1**	**11.5**	**6.7**	**3**

Table S1

Baseline demographic and clinical characteristics of the healthy volunteers. The degree of handedness was assessed by the Edinburgh Handedness Inventory, Smoking habbits refer to number of cigarettes consumed per day, alcohol consumption refers to number of units consumed per week, caffeine consumption refers to number of cups consumed per day. BMI: body mass index

I

ITEM	Positive affect	Negative affect	**Communality**
**Energetic**	0.92[a]	- 0.15	0.87
**Enthusiastic**	0.85 [a]	- 0.27	0.79
**Happy**	0.69 [a]	- 0.46 [a]	0.69
**Alert**	0.85 [a]	- 0.16	0.75
**Irritable**	- 0.43 [a]	0.63 [a]	0.58
**Sad**	- 0.17	0.86 [a]	0.74
**Stressed**	- 0.39	0.68 [a]	0.63
**Unmotivated**	- 0.72 [a]	0.25	0.58
**Upset**	- 0.15	0.89 [a]	0.81

Rotated factor loadings (VASQoM)
[a] factor loadings retained

II

	**Coefficient**	**p-value**	**95% CI**
SCC positive affect	-.011	.03	-.02 to -.01
SCC negative affect	.001	.03	.01 to .02
SCP positive affect	.219	.02	.01 to .03
SCP negative affect	-.011	<.01	-.02 to -.01
PO positive affect	.002	.59	-.01 to .01
PO negative affect	.001	.91	-.01 to .01

Effects of time on VASQoM ratings over 24 hours, data from days 3-4

III

	Coefficient	p-value	95% CI
DAY 3 positive affect	-.011	.99	-.03 to .01
DAY 3 negative affect	.009	.14	-.01 to .02
DAY 4 positive affect	-.010	.15	-.02 to .01
DAY 4 negative affect	.010	.10	-.01 to .02

Effects of time on VASQoM ratings according to day, SCC

	Coefficient	p-value	95% CI
DAY 3 positive affect	.010	.25	-.01 to .02
DAY 3 negative affect	-.014	.05	-.03 to .01
DAY 4 positive affect	.016	.04	.01 to .03
DAY 4 negative affect	-.009	.03	-.02 to -.01

Effects of time on VASQoM ratings according to day, SCP

	Coefficient	p-value	95% CI
DAY 3 positive affect	-.007	.34	-.02 to .01
DAY 3 negative affect	.009	.07	-.01 to .02
DAY 4 positive affect	.011	.04	.01 to .02
DAY 4 negative affect	-.008	.07	-.02 to .01

Effects of time on VASQoM ratings according to day, PO

Table S2

(I) Exploratory factor analysis was performed using principal component analysis to reduce the 9 items of the VASQoM (see supplementary Figure 3) to a lower number of variables and to identify empirically related groups of variables. We extracted two factors, positive affect and negative affect, based on the examination of the eigenvalues, the scree plot and the interpretability of the factors. We applied a varimax rotation to the factor loading matrix to achieve a simpler loading pattern. Only rotated factor loadings with a magnitude of 0.4 or greater were retained for the computation of the factor scores. The factor scores are a weighted sum of the loaded factors for each participant.

(II) An individual analysis of each treatment condition over the course of the 24-hour cycle revealed a decrease of positive affect ratings on the SCC, an increase in positive affect ratings on SCP and no considerable change of positive affect ratings on PO. The opposite pattern was found for negative affect ratings. The diurnal variation of mood [positive affect minus negative affect] is plotted in Figure 2.

(III) For SCC, no individual effect of day was found on negative or positive affect ratings. For SCP, negative affect started decreasing on day 3 and continued to do so on day 4, while positive affect ratings increased on day 4. For PO, there was some indication for the increase of negative affect ratings on days 3 and a decrease on day 4. There was also an increase of positive affect ratings on day 4.

CI: confidence intervals, PO: oral treatment, SCC: subcutaneous continuous infusion, SCP: subcutaneous pulsatile infusion, VASQoM: visual analogue scale questions of mood

ROIs	MNI152	DETAILS
**RAmy**	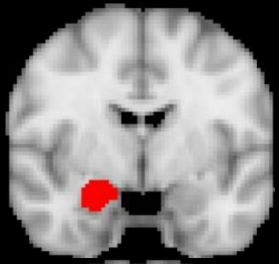	All voxels of higher probability of 50% of belonging to the ROI in the MNI152 standard space based on the Harvard-Oxford subcortical atlas.
**RNA**	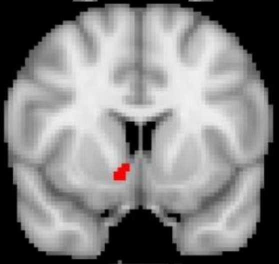	All voxels of higher probability of 50% of belonging to the ROI in the MNI152 standard space based on the Harvard-Oxford subcortical atlas.
**ROFC**	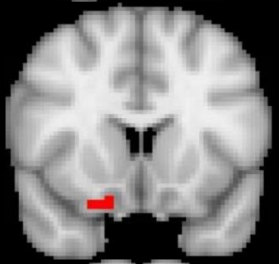	All voxels of higher probability of 80% of belonging to the ROI in the MNI152 standard space based on the Harvard-Oxford cortical atlas.
**RHipp**	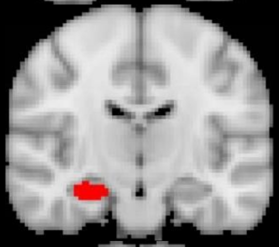	All voxels of higher probability of 70% of belonging to the ROI in the MNI152 standard space based on the Harvard-Oxford subcortical atlas.
**RCau**	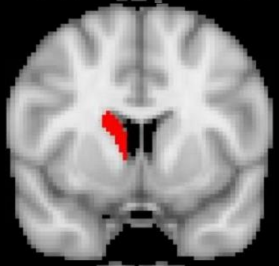	All voxels of higher probability of 50% of belonging to the ROI in the MNI152 standard space based on the Harvard-Oxford subcortical atlas.
**RPut**	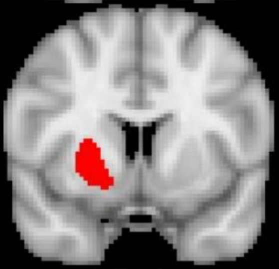	All voxels of higher probability of 50% of belonging to the ROI in the MNI152 standard space based on the Harvard-Oxford subcortical atlas.
**RAI**	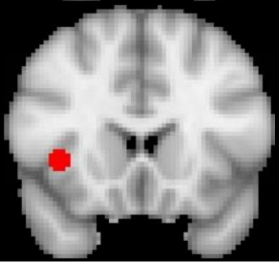	Sphere of 5 mm diameter with its centre coordinates (x/y/z) being (38/18/0) in MNI152 standard space. The sphere centre coordinates were chosen based on the anterior insula probability data (z-score) provided in neurosynth.org as an automated meta-analysis of 502 studies.

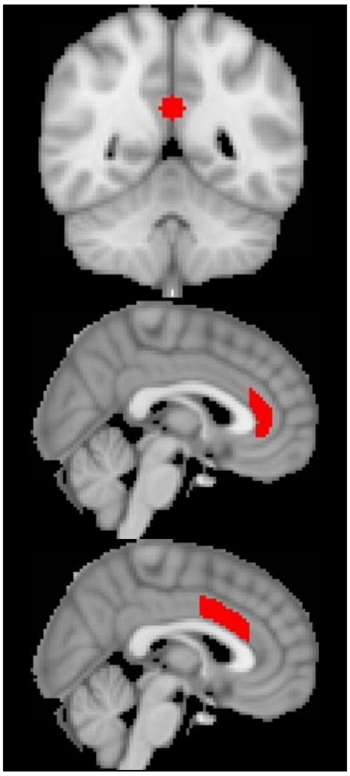

**PCC**	Sphere of 6 mm diameter with its centre coordinates (x/y/z) being (0/-50/26) in MNI152 standard space. The sphere centre coordinates were chosen based on the posterior cingulate probability data (z-score) provided in neurosynth.org as an automated meta-analysis of 726 studies.
**vACC**	All voxels of higher probability of 70% of belonging to the ventral part of anterior cingulate in the MNI152 standard space based on the Harvard-Oxford subcortical atlas.
**sACC**	All voxels of higher probability of 70% of belonging to the middle part of anterior cingulate in the MNI152 standard space based on the Harvard-Oxford subcortical atlas.

Table S3

The 10 preselected regions of interest (ROIs). In large brain regions (like the hippocampus, anterior cingulate and orbitofrontal cortex), a higher than 50% probability threshold was applied to reduce the size of the seed mask created. In relation to the division of anterior cingulate between its ventral and middle part, we chose the vertical plane through anterior boundary of the genu of the corpus callosum, and between its middle and dorsal part, the vertical plane through the anterior commissure as proposed by Yan et al. (2009).

MNI152: standard brain (by the Montreal Neurological Institute)

PCC: posterior cingulate, RAI: right anterior insula, RAmy: right amygdala, RCau: right caudate, RHipp: right hippocampus, RNA: right nucleus accumbens, ROFC: right orbitofrontal cortex, RPut: right putamen, sACC: middle part of the anterior cingulate, vACC: ventral part of the anterior cingulate

Group	ROIs	Correlation	Cluster(s)	Z-max	X	Y	Z	Brain regions
SCC	RAmy							
	RNA	Negative (p = 0.027)	482	3.81	20	36	42	R. Frontal pole, superior frontal gyrus
				3.43	8	46	24	R. Paracingulate, superior frontal gyrus
				3.08	-6	38	46	L. Superior frontal gyrus
	ROFC	Positive (p = 0.014)	395	3.68	-42	10	-24	L. Temporal pole
				3.60	-32	18	-22	L. Orbitofrontal cortex
				3.18	-40	18	-30	L. Temporal pole
SCP	RAmy	Negative (p < 0.001)	1700	3.62	34	18	-8	R. Insula
				3.53	8	38	8	ACC
				3.50	16	22	-6	R. Caudate
	RNA	Negative (p < 0.001)	827	3.84	-40	40	-14	L. Frontal pole
				3.79	-50	24	8	L. Inferior frontal gyrus
				3.75	-46	36	2	L. Frontal pole, inferior frontal gyrus
				3.70	-48	20	4	L. Inferior frontal gyrus
				3.55	-54	30	6	L. Inferior frontal gyrus
	ROFC	Negative	4058 (p < 0.001)	4.2	0	-92	2	Occipital Pole
				4.18	30	-80	44	R. Lateral occipital cortex (superior)
				4.16	2	-72	42	Precuneous
				4.12	32	-76	48	R. Lateral occipital cortex (superior)
				4.04	-48	-70	42	L. Lateral occipital cortex (superior)
			1532 (p < 0.001)	3.59	-44	28	-4	L. Orbitofrontal cortex, inferior frontal gyrus, f. operculum
				3.59	-46	36	6	L. Inferior frontal gyrus, Frontal pole
				3.59	-48	22	28	L. Middle frontal gyrus
				3.48	-42	40	0	L. Frontal Pole
				3.47	-54	16	0	L. Inferior frontal gyrus
				3.46	-44	-18	22	L. c. operculum
				3.45	-26	8	8	L. Putamen

				3.41	-36	-12	20	L. c. operculum
				3.33	-36	-10	16	L. c. operculum, insula
			659 (p < 0.001)	3.59	50	24	28	R. Middle frontal gyrus
				3.28	44	20	20	R. Inferior frontal gyrus
				3.2	50	14	12	R. Inferior frontal gyrus
				3.12	52	26	10	R. Inferior frontal gyrus
				3.08	56	8	8	R. Precentral gyrus, Inferior frontal gyrus
				3.05	40	20	26	R. Middle frontal gyrus, inferior frontal gyrus
			572 (p = 0.001)	4.09	4	-32	50	Precentral gyrus, PCC
				4.02	0	-30	44	PCC
				3.74	4	-40	56	Precuneous, postcentral gyrus
				3.41	-4	-36	44	PCC, precuneous
				3.37	-2	-38	54	Precentral gyrus, postcentral gyrus, precuneous
				3.34	0	-50	50	Precuneous
			348 (p = 0.028)	3.51	42	6	6	R. c. operculum
				3.45	32	-6	8	R. Putamen
				3.21	32	-18	10	R. insula
				3.04	30	6	10	R. putamen, insula
PO	RAmy	[redacted]						
	RNA							
	ROFC	Negative	416 (p = 0.013)	3.71	-6	-70	2	L. Lingual gyrus
				3.68	18	-64	20	R. Supracalcarine cortex, cuneal cortex, precuneus
				3.37	-6	-72	16	L. Intracalcarine cortex, cuneal-, supracalcarine cortex
				3.34	6	-76	16	R. Supracalcarine cortex, intracalcarine -, cuneal cortex
				3.26	10	-70	16	R. Intracalcarine cortex
				3.15	-12	-72	0	L. Lingual gyrus
			341 (p = 0.040)	3.69	-58	4	30	L. Precentral gyrus
				3.67	-56	0	26	L. Precentral gyrus

(part A)

Treatment mode	ROIs	Correlated neural activity in following brain regions
SCC	RAmy	-
	RNA	R. Frontal pole, R. paracingulate, R./L. Superior frontal gyrus
	ROFC	L. Temporal pole, L. Orbitofrontal cortex
SCP	RAmy	R. Insula, ACC, R. Caudate (**salience network**)
	RNA	L. Frontal pole, L. inferior frontal gyrus
	ROFC	Occipital pole, Precuneous, L./R. superior LOC
		L. Operculum, L. Insula, L. Putamen, L. OFC, L. Inferior frontal gyrus, L. Frontal pole (**salience network/ ECN**)
		R. Middle frontal gyrus, R. Inferior frontal gyrus, R. precentral gyrus (**executive control network**)
		PCC, Precuneous, Pre- & Postcentral gyrus (**default mode network**)
		R. Central operculum, R. Insula, R. Putamen (**salience network**)
PO	RAmy	-
	RNA	-
	ROFC	L. Lingual gyrus, L./R. Intracalcarine & Supracalcarine & Cuneal cortex, R. Precuneous
		L. Precentral gyrus

(part B)

Table S4

(part A) Details on the statistical significance and the neuroanatomical maxima of the clusters, which form functional (correlated or anticorrelated) networks with the three regions of interest (ROIs), correlating with positive affect variation per treatment group.

(part B) Summary table of part A. In red, brain regions whose neural activity negatively correlates with corresponding ROI. In green, brain regions whose neural activity negatively correlates with corresponding ROI.

L.: left, PO: oral treatment group, R.: right, RAmy: right amygdala, RNA: right nucleus accumbens, ROFC: right orbitofrontal cortex, SCC: subcutaneous-continuous treatment group, SCP: subcutaneous-pulsatile treatment group.
